# Engineering Biodegradable and Biocompatible Bio-ionic Liquid Conjugated Hydrogels with Tunable Conductivity and Mechanical Properties

**DOI:** 10.1038/s41598-017-04280-w

**Published:** 2017-06-28

**Authors:** Iman Noshadi, Brian W. Walker, Roberto Portillo-Lara, Ehsan Shirzaei Sani, Nayara Gomes, Mohammad Reza Aziziyan, Nasim Annabi

**Affiliations:** 1 Department of Chemical Engineering, Northeastern University, Boston, MA 02115 USA; 2 Harvard-MIT Division of Health Sciences and Technology, Massachusetts Institute of Technology, Cambridge, MA 02139 USA; 3 Biomaterials Innovation Research Center, Brigham and Women’s Hospital, Harvard Medical School, Boston, MA USA; 4 Centro de Biotecnología-FEMSA, Tecnológico de Monterrey, Monterrey, NL, 64700 México, USA; 5 Interdisciplinary Institute for Technological Innovation (3IT), CNRS UMI-3463, Université de Sherbrooke, 3000 boul. de l’Université, Sherbrooke, Québec J1K 0A5 Canada; 6 Department of Electrical and Computer Engineering, Faculty of Engineering, Université de Sherbrooke, 2500 boul. de l’Université, Sherbrooke, Québec J1K 2R1 Canada

## Abstract

Conventional methods to engineer electroconductive hydrogels (ECHs) through the incorporation of conductive nanomaterials and polymers exhibit major technical limitations. These are mainly associated with the cytotoxicity, as well as poor solubility, processability, and biodegradability of their components. Here, we describe the engineering of a new class of ECHs through the functionalization of non-conductive polymers with a conductive choline-based bio-ionic liquid (Bio-IL). Bio-IL conjugated hydrogels exhibited a wide range of highly tunable physical properties, remarkable *in vitro* and *in vivo* biocompatibility, and high electrical conductivity without the need for additional conductive components. The engineered hydrogels could support the growth and function of primary cardiomyocytes in both two dimentinal (2D) and three dimensional (3D) cultures *in vitro*. Furthermore, they were shown to be efficiently biodegraded and possess low immunogenicity when implanted subcutaneously in rats. Taken together, our results suggest that Bio-IL conjugated hydrogels could be implemented and readily tailored to different biomedical and tissue engineering applications.

## Introduction

Hydrogels are three dimensional (3D) polymeric networks made from hydrophilic, natural or synthetic polymers, which are rendered insoluble by using different types of crosslinking mechanisms^1^. They are widely used for several biomedical applications due to their resemblance to the native extracellular matrix (ECM), as well as their remarkable biocompatibility, and tunable mechanical and biochemical properties^2, 3^. However, hydrogels are typically non-conductive, which limits their application as bioactive scaffolds for excitable tissues, such as neural, as well as cardiac, and skeletal muscle tissues^4^. More recently, hydrogels with electroconductive properties have been developed through the incorporation of different nanomaterials (e.g. gold nanoparticles, silver nanowires, carbon nanotubes, graphene oxide (GO)) and conductive polymers (e.g. polyaniline, polypyrrole (PPy), polythiophene) to their network^5–11^. These electroconductive hydrogels (ECHs) constitute a class of smart biomaterials that combine the electrical properties of intrinsically conductive elements, with highly hydrated, and biocompatible hydrogel networks^1^.

Due to the responsiveness of several tissues to electrical stimulation, the use of ECHs and conductive polymers has been explored for various biomedical applications, such as electrochemical biosensors^12–14^, and electro-responsive drug delivery systems^15–17^. In addition, ECHs have been extensively used as tissue engineered scaffolds to promote cellular adhesion, proliferation, differentiation, and growth, as well as the electroactive modulation of different cell types, including neurons, cardiomyocytes (CMs), cardiac fibroblasts (CFs), preosteoblasts, endothelial cells, and mesenchymal stem cells (MSCs)^18–23^. As a materials platform for tissue engineering, the physical properties of ECHs, such as their mechanical properties, water uptake capability, porosity, and degradation rate can influence tissue regeneration *in vivo* ^24, 25^. In addition, excessive water uptake has been shown to impair the conductivity of ECHs, due to the percolation of fluids across the polymer network^23^. Therefore, several approaches have been used to modulate the conductivity and physical properties of ECHs, including the incorporation of different base polymers, bioactive components, porogens, and hydrophilic or hydrophobic moieties, as well as variations on the degree of crosslinking within the polymer networks^26–28^.

Despite the wide application of ECHs and conductive polymers for biomedical applications, they still exhibit major technical limitations, including the absence of cell binding sites, cytotoxicity, as well as poor solubility, processability, and biodegradability^5–11^. In addition, conventional strategies used to synthesize ECHs often suffer from difficult and prolonged processing, harsh reaction conditions, and difficulties in the incorporation of the conductive materials, and modulation of their physical properties. For example, we have previously reported the incorporation of GO nanoparticles into methacryloyl-substituted tropoelastin (MeTro) hydrogels for cardiac tissue engineering^29^. Although the resulting scaffolds could support the function of primary CMs *in vitro*, the ability to finely tune their mechanical properties and conductivity was limited. In addition, the appropriate dispersion of conductive nanomaterials in polymeric matrices remains technically challenging^30–32^. Other studies have reported that conductive polymers such as polyaniline, are associated with increased cytotoxicity and overexpression of pro-inflammatory cytokines *in vitro* ^33, 34^. Therefore, there is an unmet need for the development of biocompatible, biodegradable, and electroconductive biomaterials with highly tunable electrical and physical properties.

Ionic liquids (ILs) are organic salts with a low melting point and high water solubility, as well as high ionic conductivity and electrochemical stability^35^. Recently, ILs have emerged as promising alternatives in the field of material synthesis, due to their high thermal stability, ionic conductivity, and electrochemical stability^36^. Among the variety of ILs, choline-based bio-ionic liquids (Bio-ILs) have gained much interest due to their enhanced biocompatibility^35, 37^. Choline is a precursor of the phospholipids that comprise biological cell membranes in mammalian and plant tissues, such as phosphatidylcholine and sphingomyelin. In addition, previous studies have shown that choline can be decomposed both physiologically and environmentally to smaller chain molecules^38^. Consequently, choline-based Bio-ILs have been extensively investigated as non-toxic components for numerous applications^39–41^. Unlike conventional ILs, Bio-ILs are biodegradable and non-cytotoxic, as they are comprised solely of naturally derived compounds^35^. Bio-ILs have been used as biocompatible materials for various applications, such as multi-responsive drug delivery systems^42^, solvents for biopolymers^43^, as well as ion gels for sensors and actuators^44^. In the present work, we introduce a new class of photocrosslinkable ECHs, through the conjugation of different polymers with a conductive choline-based Bio-IL. This approach provides intrinsic conductivity to the polymer network, without the need for additional electroconductive components. ECHs with different mechanical and electroconductive profiles were generated through Bio-IL conjugation of two widely used photocrosslinkable polymers (i.e., gelatin methacryloyl (GelMA) and poly(ethylene glycol) diacrylate (PEGDA)). In contrast to conventional methods based on ultraviolet (UV)-activated photocrosslinking, Bio-IL conjugated hydrogels with tunable conductivity and mechanical properties were rapidly formed via exposure to visible light. We assessed the ability of the composite hydrogels to promote the adhesion, proliferation, and electromodulation of primary CMs *in vitro*. We also evaluated the capability of Bio-IL conjugated hydrogels to propagate electrical stimuli and restore synchronous contraction in severed skeletal muscle *ex vivo*. We also investigated the biodegradability and immunogenicity of the engineered ECHs via subcutaneous implantation in an animal model. This new class of electroconductive polymer/Bio-IL hydrogel system holds remarkable potential for tissue engineering applications, due to their tunable properties, as well as their *in vitro* and *in vivo* biocompatibility and biodegradability. In addition, due to their intrinsic electroconductive properties they can also be used to engineer flexible and stretchable electronics for biomedical applications, including epidermal sensors, smart sutures, artificial electronic muscles, neuron-to-machine interfaces, and implantable medical devices. Furthermore, the approach described here can potentially be used to engineer ECHs from different photocrosslinkable polymers such as collagen, elastin-like polypeptides, tropoelastin, hyaluronic acid, alginate, polyvinyl alcohol (PVA), polycaprolactone (PCL), etc.

## Synthesis of Bio-IL conjugated ECHs

**Figure 1:**
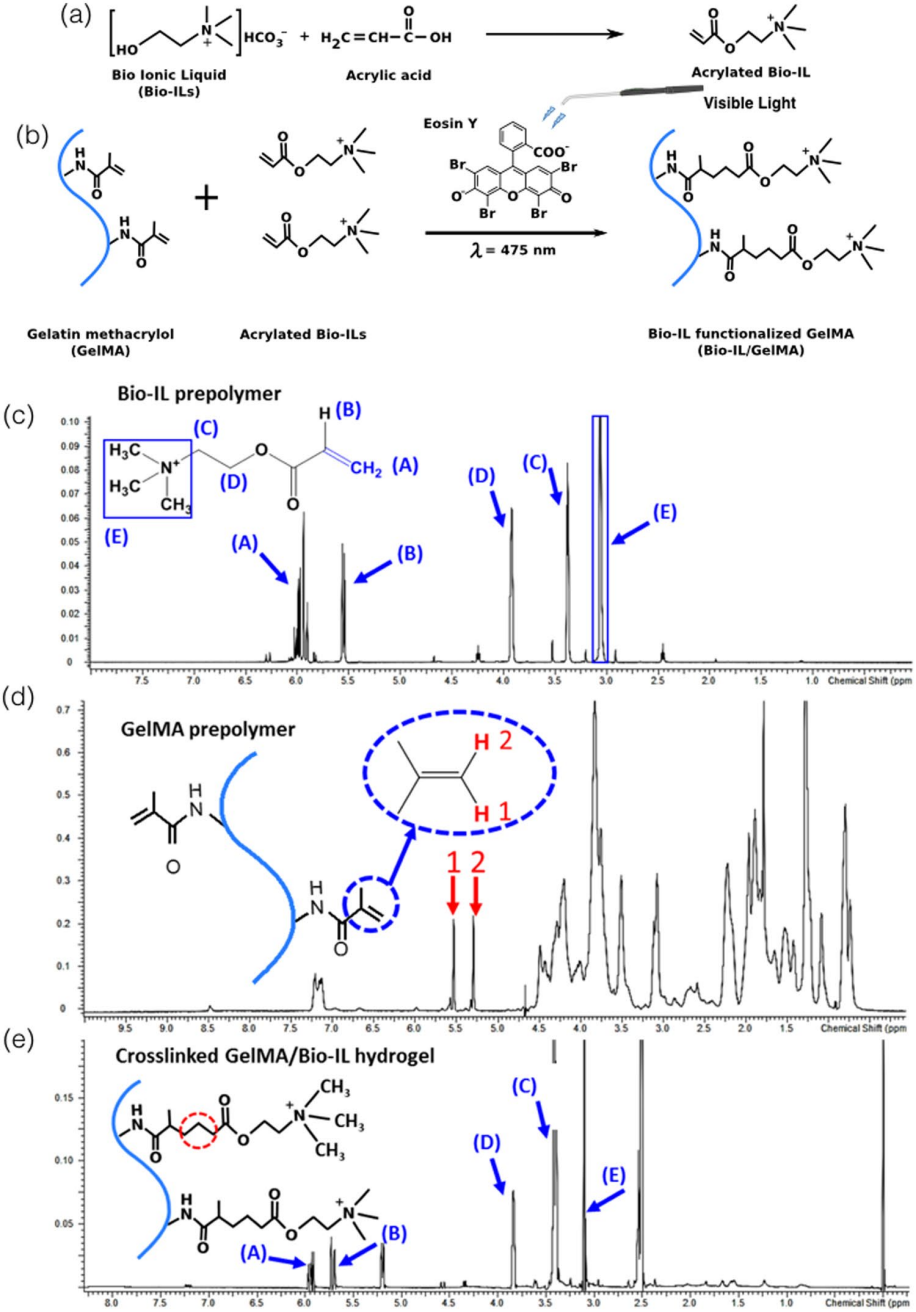
Synthesis and characterization of Bio-IL functionalized GelMA hydrogels. The panels show schematics of the proposed reactions for (**a**) the acrylation of choline bicarbonate to form Bio-IL, and (**b**) the reaction between GelMA and Bio-IL in the presence of Eosin Y and visible light to form GelMA/Bio-IL hydrogel. ^1^H-NMR﻿ analysis of (**c**) Bio-IL prepolymer, (**d**) GelMA prepolymer, and (**e**) GelMA/Bio-IL composite hydrogel. GelMA/Bio-IL hydrogels were formed by using 1% VC, 1.5% TEOA, and 0.1 mM Eosin Y at 120 s light exposure.

Herein, we describe a versatile method to conjugate choline-based Bio-ILs to both natural and synthetic polymers, to yield biodegradable and biocompatible ECHs (Figs 1 and S1). GelMA biopolymer was synthesized according to a methodology reported previously^45^. The Bio-IL was synthesized based on the reaction between choline bicarbonate and acrylic acid (Fig. 1a). Different ratios of GelMA and Bio-IL were then mixed at room temperature. The resulting GelMA/Bio-IL prepolymer was then crosslinked into a hydrogel via visible-light initiated photopolymerization, using Eosin Y, vinyl caprolactone (VC), and triethanolamine (TEOA) (Fig. 1b). Composite hydrogels were synthesized using 100/0 (control), 80/20, 50/50, and 20/80 polymer/Bio-IL ratios at 10%, 15% and 20% (w/v) final polymer concentrations.

The acrylation of choline bicarbonate was confirmed by comparing the proton nuclear magnetic resonance (^1^H NMR) spectra of choline bicarbonate with that of the choline acrylate (Bio-IL) as shown in Figure S2. The appearance of a peak related to the hydrogen atoms in the acrylate groups at δ = 5.8–6.1 ppm was indicative of the acrylation of choline bicarbonate (Figs 1c and S1c). In addition, the ^1^H NMR spectra were collected for GelMA prepolymer (Fig. 1d), and GelMA/Bio-IL composite hydrogel (Fig. 1e) to confirm the conjugation of Bio-IL to GelMA. We used Equation 1 to calculate the continuous decrease of the C=C double bond signal in the GelMA methacrylate groups after exposure to visible light. By comparison between ^1^H NMR spectra of GelMA prepolymer (Fig. 1d) and GelMA/Bio-IL composite hydrogel (Fig. 1e), it was found that 94.1 ± 4.6 % of the methacrylate groups in GelMA/Bio-IL composites disappeared after photocrosslinking. In addition, 57.4 ± 4.3 % of the peak area related to C=C double bonds of the acrylate groups in Bio-IL (Fig. 1c) also disappeared in composite GelMA/Bio-IL hydrogels following crosslinking (Fig. 1e). This can confirm the incorporation of both Bio-IL and GelMA in the resulting composite hydrogel. The appearance of a sharp peak at δ = 3.1–3.2 ppm in the composite hydrogel (Fig. 1e), corresponding to the three hydrogen atoms of choline (ammonium ion), could also confirm the conjugation of Bio-IL to the hydrogel network. This peak was absent in the GelMA prepolymer spectrum (Fig. 1d), but it was observed in both the Bio-IL (Fig. 1c) and the composite GelMA/Bio-IL hydrogel (Fig. 1e). Similarly, as shown in Figure S1, the choline peaks (d = 3.1–3.2 ppm) were also observed in PEGDA/Bio-IL hydrogels, indicating the conjugation of Bio-IL to PEGDA.

## Characterization of the electroconductive properties of engineered ECHs

**Figure 2:**
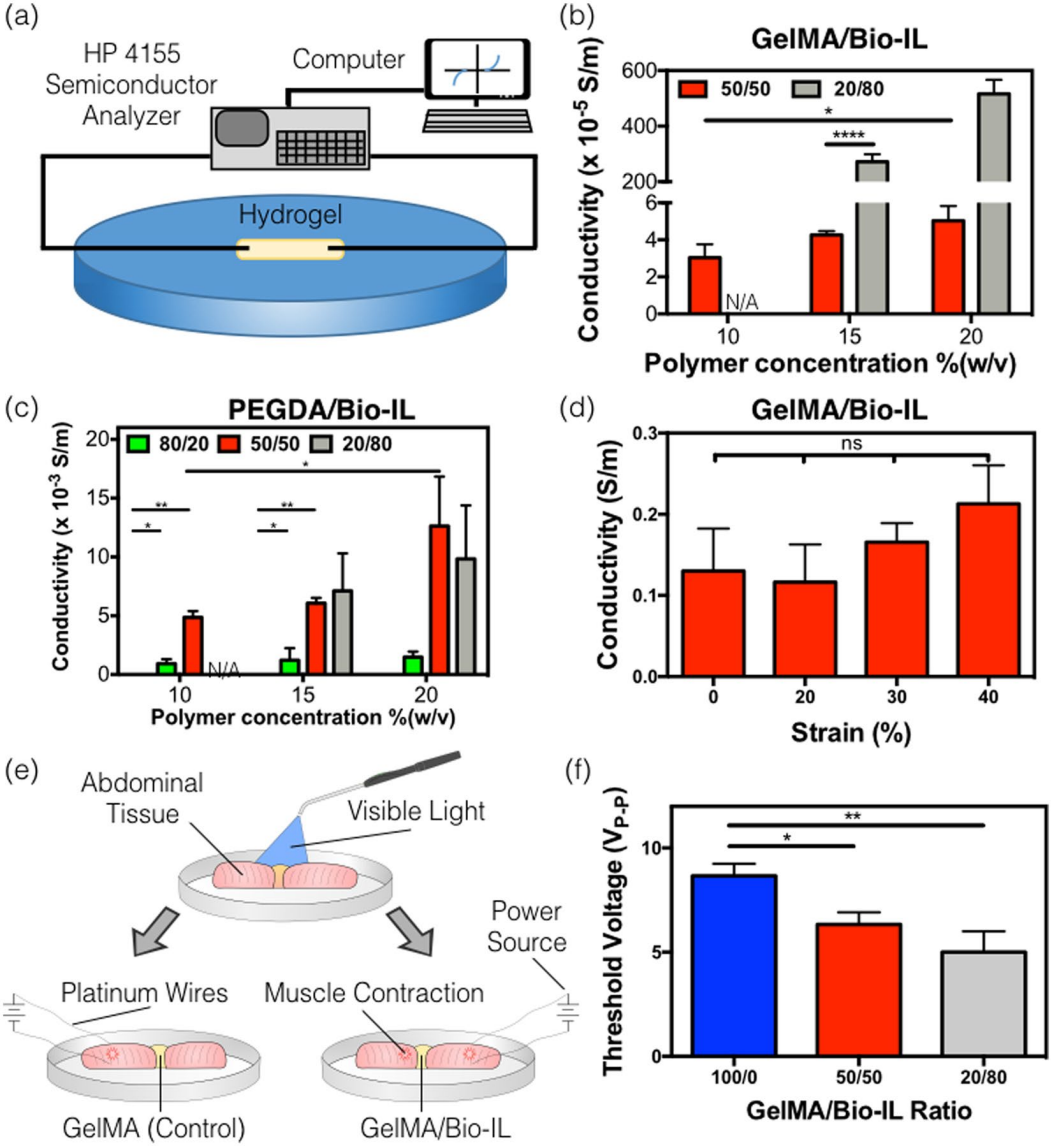
Electrical conductivity of the engineered polymer/Bio-IL hydrogels crosslinked by visible light. (**a**) Experimental set-up of the two-probe electrical station used to measure the electrical conductivity of the engineered hydrogels. Conductivity measurements of Bio-IL conjugated (**b**) GelMA and (**c**) PEGDA hydrogels at different polymer concentrations and polymer/Bio-IL ratios (1% VC, 1.5% TEOA, 0.1 mM Eosin Y, and 120 s exposure to visible light were used to form GelMA and PEGDA hydrogels). (**d**) Electrical conductivity of 15% GelMA/Bio-IL composite hydrogels at 50/50 ratio, which was stretched up to 0%, 20%, 30%, and 40% strain level demonstrating no significant changes in conductivity after stretching. (**e**) Schematic of *ex vivo* experiments performed using rat abdominal muscle tissues, connected using 15% final polymer concentration and 50/50 GelMA/Bio-IL ratio, and pure GelMA hydrogels (**f**) Threshold voltages at which contraction was achieved using 15% final polymer concentration GelMA/Bio-IL at 50/50 and 20/80 ratios, as well as pure 15% GelMA hydrogels. Error bars indicate standard error of the means, asterisks mark significance levels of p < 0.05 (*), p < 0.01 (**), and p < 0.001 (***).

Conventional polymer-based hydrogels, including those based on GelMA and PEGDA, are intrinsically non-conductive. This characteristic limits their application for the modulation of excitable cell types, such as neurons and CMs. Therefore, we aimed to determine if the conjugation of a choline-based Bio-IL could provide electroconductive properties to these polymer-based hydrogels. Briefly, Bio-IL functionalized GelMA and PEGDA hydrogels were synthesized as described before, and allowed to dry for 24 h. In particular, we could not form stable hydrogels with 20/80 polymer/Bio-IL ratios at 10% final polymer concentration. This was likely due to the low concentration of polymer within the network. The partially dried hydrogels were placed in a two-probe electrical station connected to a Hewlett Packard 4155A Semiconductor Parameter analyzer to measure their conductivity (Fig. 2a). A probe was placed at each end of the hydrogels and voltage was applied in increments of 0.05 V, from −25 to 25 V. The variations in the current were recorded, and the conductivity was calculated using Ohm’s Law^46^. Our results demonstrated that the use of different final polymer concentrations, as well as different polymer/Bio-IL ratios enabled the modulation of the electrical properties of the composite ECHs. For instance, the conductivity of 50/50 GelMA/Bio-IL hydrogels increased from 3.03 × 10^−05^ ± 0.72 × 10^−05^ S/m to 4.27 × 10^−05^ ± 0.21 × 10^−05^ S/m, and 5.03 × 10^−05^ ± 0.80 × 10^−05^ S/m, when the final polymer concentration was increased from 10% to 15% and 20%, respectively (Fig. 2b). Furthermore, the conductivity of 15% GelMA/Bio-IL hydrogels increased more than 63-fold, from 4.27 × 10^−05^ ± 0.21 × 10^−05^ S/m to 272 × 10^−05^ ± 27.05 × 10^−05^ S/m, when the GelMA/Bio-IL ratio was changed from 50/50 to 20/80 (Fig. 2b).

Similar to what we observed for GelMA-based hydrogels, the conductivity of 50/50 PEGDA/Bio-IL hydrogels also increased from 485 × 10^−05^ ± 54.93 × 10^−05^ S/m to 608 × 10^−05^ ± 44.85 × 10^−05^ S/m and 1262 × 10^−05^ ± 421 × 10^−05^ S/m, when the final polymer concentration was increased from 10% to 15% and 20%, respectively (Fig. 2c). In addition, the conductivity of 15% PEGDA/Bio-IL hydrogels was also increased from 121 × 10^−05^ ± 1.01 × 10^−05^ S/m to 608 × 10^−05^ ± 44.85 × 10^−05^ and 711 × 10^−05^ ± 320 × 10^−05^ S/m, when the PEGDA/Bio-IL ratio was changed from 80/20 to 50/50 and 20/80, respectively (Fig. 2c). Interestingly, hydrogels with 80/20 GelMA/Bio-IL ratios did not exhibit any apparent conductivity in our experiments (Fig. 2b). However, PEGDA-based hydrogels with the same polymer/Bio-IL ratio did show measurable levels of conductivity, at all final polymer concentrations tested (Fig. 2c). These observations suggest that the use of different polymers could also influence the degree of conductivity of Bio-IL conjugated hydrogels. Taken together, these results demonstrated that the conductivity of the engineered ECHs increased consistently by increasing the concentrations of Bio-IL (Fig. 2b and c). In particular, the maximum conductivity observed in our tests corresponded to 516 × 10^−05^ ± 50 × 10^−05^ S/m and 1,262 × 10^−05^ ± 420 × 10^−05^ S/m, for GelMA- and PEGDA-based hydrogels, respectively (Fig. 2b and c). These results suggest that the 20/80 GelMA/Bio-IL formulation, which exhibited a conductivity of 516 × 10^−05^ S/m, could potentially be implemented in cardiac tissue engineering applications, since the conductivity of the native myocardium has been shown to be approximately 500 × 10^−05^ S/m^47, 48^.

The engineering of elastic and conductive materials that retain their electrical properties under substantial stretching and bending, still constitutes a major technical challenge^49, 50^. Therefore, we aimed to investigate the conductivity of the Bio-IL conjugated hydrogels at different levels of stretching. No conductivity measurements at different strain levels could be obtained from PEGDA/Bio-IL hydrogels, since the samples broke after applying stretch at all polymer and Bio-IL concentrations tested. Hence, we focused our analysis solely on GelMA-based hydrogels. Briefly, GelMA/Bio-IL hydrogels were dried for 2 h to retain trace amounts of moisture, and maintain their flexibility. The samples were then stretched and the conductivity was measured at different strain levels, using a two-probe electrical station. These results showed that there were no statistically significant differences between the conductivity of hydrogels at different levels of stretching (Fig. 2d). In contrast, alternative methods to engineer ECHs, such as the incorporation of dispersed metallic nanoparticles, often suffer from a rapid and nonlinear decrease in conductivity during stretching^49, 51, 52^.

One of the most widely explored applications of ECHs is their use as conductive scaffolds for engineering excitable tissue constructs. Thus, we evaluated the potential of Bio-IL conjugated polymers to restore the propagation of electrical stimuli across severed skeletal muscle tissues *ex vivo*. Briefly, the rectus abdominis muscles of female Wistar rats were explanted after euthanasia, and cut into square pieces. The tissues were placed 3 mm apart, in an electrically insulated polydimethylsiloxane (PDMS) mold. GelMA/Bio-IL hydrogels, as well as pure GelMA controls were photocrosslinked *in situ*, between the two pieces of tissue (Fig. 2e). Pulsed direct current test runs were conducted by applying 50 ms square pulses at increasing frequencies, using short platinum wires that were positioned on one of the two pieces of muscle tissue. The induction of contraction was visually inspected in the sample on the opposite end of the hydrogel, after applying electrical pulses at increasing voltages (Supplementary Video 1). These results demonstrated that muscle tissue samples joined together using GelMA/Bio-IL hydrogels exhibited a significantly lower excitation threshold, when compared to pure GelMA controls (Fig. 2f). These observations suggest that Bio-IL functionalized polymers could be used to restore functional integrity, in tissues in which electrophysiological communication has been interrupted. Previous works have demonstrated the ability of ECHs to restore electrophysiological coupling of severed skeletal muscle tissue *ex vivo*, using PPy-chitosan^53^ and GO/MeTro hydrogels^29^. However, the incorporation of the PPy polymer, as well as GO nanoparticles into the hydrogel networks was associated with poorly tunable conductive properties. In contrast, Bio-IL functionalized hydrogels exhibited a wide range of electroconductive properties (Fig. 2b and c).

## Characterization of the mechanical properties of engineered ECHs

**Figure 3:**
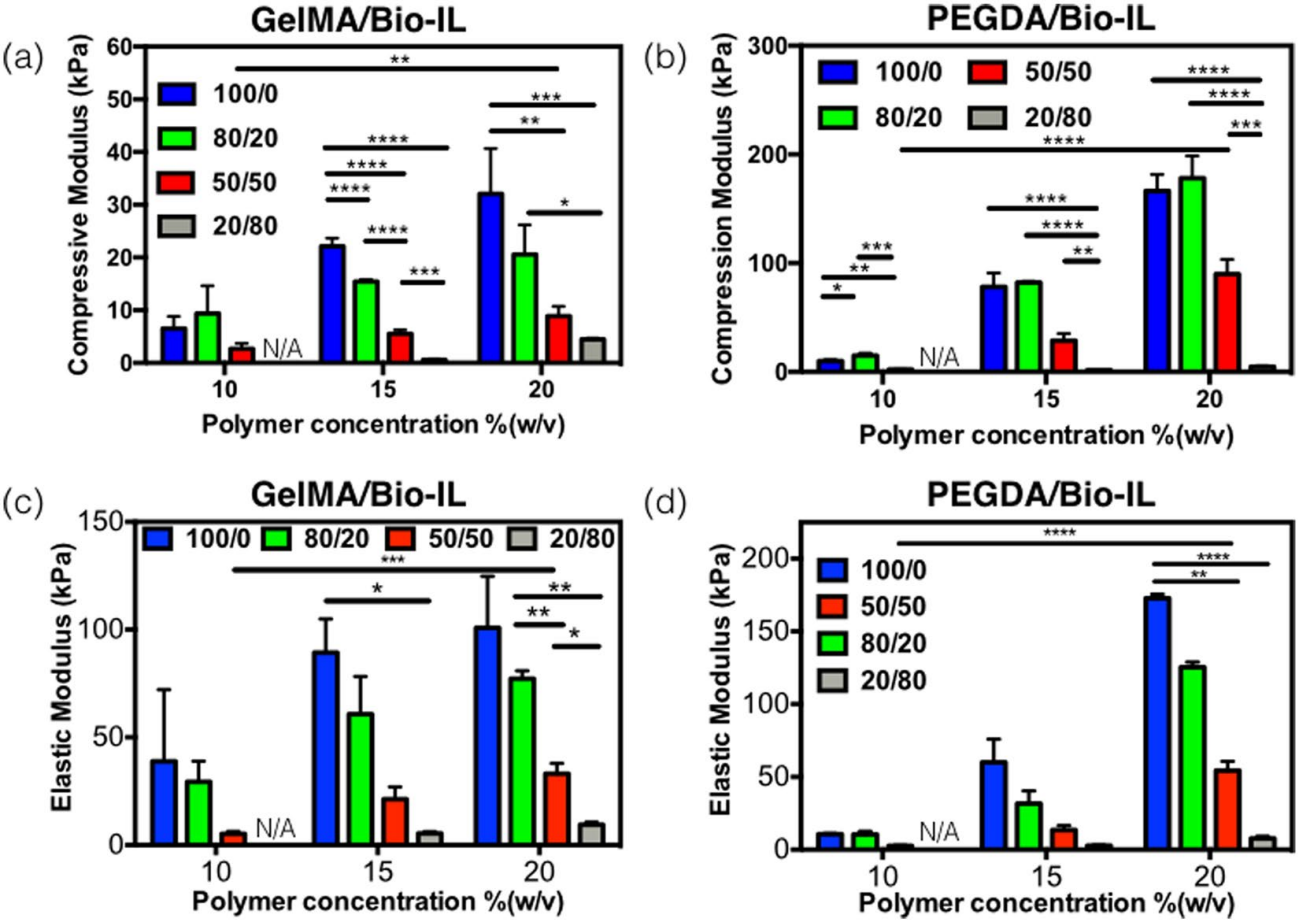
Mechanical properties of Bio-IL conjugated GelMA and PEGDA hydrogels crosslinked with visible light. Compressive moduli for (**a**) GelMA/Bio-IL and (**b**) PEGDA/Bio-IL hydrogels engineered by varying polymer concentration and polymer/Bio-IL ratios. Elastic moduli for (**c**) GelMA/Bio-IL and (**d**) PEGDA/Bio-IL hydrogels with varying polymer concentration and polymer/Bio-IL ratios (1% VC, 1.5% TEOA, 0.1 mM Eosin Y, and 120 s light exposure were used to form GelMA and PEGDA hydrogels). Error bars indicate standard error of the means, asterisks mark significance levels of p < 0.05 (*), p < 0.01 (**), and p < 0.001 (***).

Hydrogels used in biomedical applications must provide adequate mechanical support to cells and tissues, as well as the effective transduction of physicochemical stimuli. In particular, mechanical cues are known to modulate key cellular functions such as cell proliferation, differentiation, migration, and apoptosis^54^. Here, we characterized the mechanical properties of the engineered composite hydrogels. Tensile and compression tests were performed on Bio-IL conjugated hydrogels at different polymer/Bio-IL ratios, as well as different final polymer concentrations (Figs 3, S3 and S4). Bio-IL conjugated hydrogels exhibited tunable compressive moduli in the range of 0.60 ± 0.20 kPa to 32.07 ± 8.61 kPa (Fig. 3a) and 1.27 ± 0.05 kPa to 178.13 ± 20.59 kPa (Fig. 3b) for GelMA- and PEGDA-based hydrogels, respectively. In addition, our results revealed that the compressive moduli for both GelMA- and PEGDA-based hydrogels increased concomitantly, by increasing the final polymer concentration as well as the ratio of polymer to Bio-IL. For instance, the compressive moduli of 15% GelMA/Bio-IL hydrogels were shown to increase from 0.60 ± 0.20 kPa to 22.10 ± 1.56 kPa, when the ratio of GelMA to Bio-IL was increased from 20/80 to 100/0, respectively (Fig. 3a). In addition, the compressive moduli of 50/50 GelMA/Bio-IL hydrogels were also increased from 2.65 ± 1.06 kPa to 5.53 ± 0.76 kPa and 8.87 ± 1.83 kPa, when the final polymer concentration was increased from 10% to 15% and 20%, respectively (Fig. 3a). This trend was also observed for PEGDA/Bio-IL hydrogels. However, the use of PEGDA-based hydrogels yielded significantly higher compressive moduli, as compared to GelMA-based hydrogels (Fig. 3b). For instance, the compressive moduli of 15% PEGDA/Bio-IL hydrogels were increased from 1.27 ± 0.06 kPa to 78.17 ± 12.80 kPa, when the ratio of PEGDA to Bio-IL was increased from 20/80 to 100/0, respectively (Fig. 3b). In addition, the compressive moduli of 50/50 PEGDA/Bio-IL hydrogels were also increased from 2.17 ± 0.23 kPa to 28.73 ± 6.31 kPa and 89.83 ± 13.59 kPa, when the final polymer concentration was raised from 10% to 15% and 20%, respectively (Fig. 3b).

We also performed tensile tests to measure the elastic modulus of the composite hydrogels. These exhibited a wide range of elastic moduli, in the range of 5.20 ± 1.15 kPa to 100.77 ± 23.95 kPa (Fig. 3c) and 2.60 ± 0.46 kPa to 172.70 ± 2.86 kPa (Fig. 3d) for GelMA- and PEGDA-based hydrogels, respectively. Similar to what we observed for the compressive moduli, the elastic moduli of Bio-IL conjugated hydrogels were also dependent on the final polymer concentration, as well as the ratio of polymer to Bio-IL. For instance, the elastic moduli of 15% GelMA/Bio-IL hydrogels were shown to increase consistently from 5.40 ± 0.87 kPa to 89.30 ± 15.65 kPa, when the ratio of polymer to Bio-IL was increased from 20/80 to 100/0, respectively (Fig. 3c). Furthermore, the ultimate strain of GelMA/Bio-IL hydrogels were shown to increase by increasing the concentration of Bio-IL. For example, for 15% GelMA/Bio-IL hydrogels the ultimate strain increased from 13.93 ± 4.50% to 31.25 ± 5.10% when the ratio of GelMA to Bio/IL was changed from 100/0 to 20/80, respectively (Figure S4a). However, the ultimate stress decreased by increasing the concentration of Bio-IL. As shown in Figure S4c, the ultimate stress decreased from 9.97 ± 1.42 kPa to 0.97 ± 0.25 kPa when the GelMA to Bio-IL ratio was changed from 100/0 to 20/80 at 15% final polymer concentration. In addition, the elastic moduli of 50/50 GelMA/Bio-IL hydrogels were also increased from 5.20 ± 1.15 kPa to 21.30 ± 5.75 kPa and 33.13 ± 4.78 kPa, when the final polymer concentration was increased from 10% to 15% and 20%, respectively (Fig. 3c). Once again, the use of PEGDA-based hydrogels yielded significantly higher elastic moduli, as compared to GelMA-based hydrogels (Fig. 3d). The elastic moduli of 15% PEGDA/Bio-IL hydrogels increased from 2.73 ± 0.43 kPa to 60.00 ± 15.85 kPa, when the ratio of PEGDA to Bio-IL was increased from 20/80 to 100/0, respectively (Fig. 3d). Similar to GelMA/Bio-IL hydrogels, the ultimate strain of 15% PEGDA/Bio-IL hydrogels were shown to increase from 9.28 ± 2.26% to 59.65 ± 11.59% when the ratio of PEGDA to Bio/IL was changed from 100/0 to 20/80, respectively (Figure S4b). The ultimate stress also increased from 1.53 ± 0.40 kPa for the 20/80 ratio, to 6.10 ± 3.22 kPa for the 100/0 for 15% PEGDA/Bio-IL hydrogels (Figure S4d). Lastly, the elastic moduli of 50/50 PEGDA/Bio-IL hydrogels were also increased from 2.60 ± 0.46 kPa to 13.43 ± 3.20 kPa and 54.37 ± 6.34 kPa, when the final polymer concentration was increased from 10% to 15% and 20%, respectively (Fig. 3d).

Taken together, our results revealed that the mechanical properties of the composite scaffolds could be efficiently modulated by varying both the final polymer concentration, as well as the polymer/Bio-IL ratio. The enhanced mechanical properties at higher polymer concentrations could be explained in part due to the presence of a greater number of available crosslinking sites^55^. Moreover, the engineered scaffolds present several technical advantages in the context of biomedical applications. For example, previous studies have demonstrated that the stiffness of the native human myocardium ranges from 20 kPa to 100 kPa^56, 57^. Therefore, the highly versatile and tunable mechanical properties of these biomaterials could be used to generate ECHs with varying degrees of stiffness, which will mimic the mechanical properties of the native cardiac tissues.

## Characterization of pore size, swelling ratios and ***in vitro*** degradation of engineered ECHs

**Figure 4:**
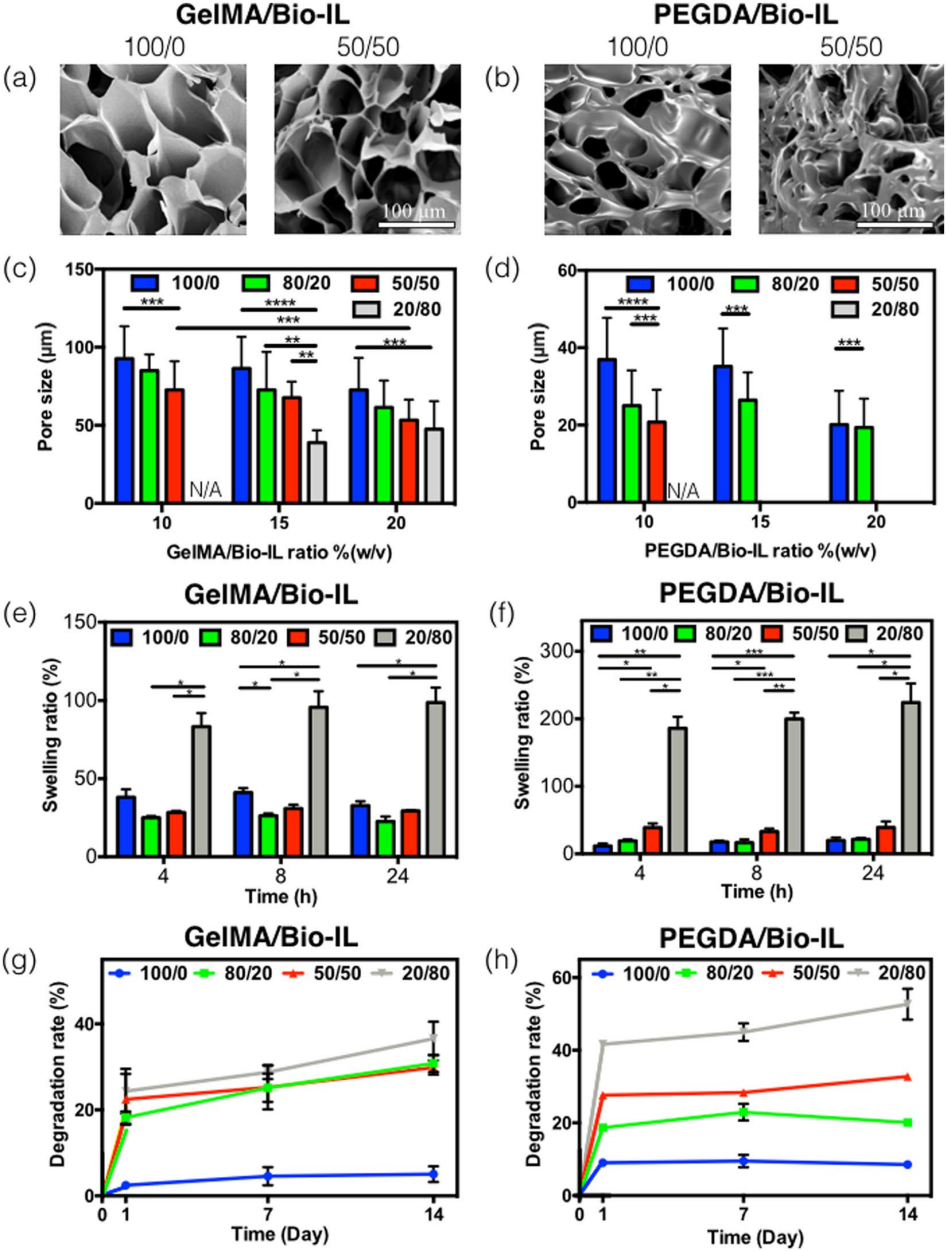
Pore characteristics, *in vitro* swelling, and degradation of polymer/Bio-IL hydrogels. Representative SEM images of (**a**) GelMA/Bio-IL and (**b**) PEGDA/Bio-IL hydrogels formed by using 100/0 and 50/50 polymer/Bio-IL ratio at 15% (w/v) polymer concentration (1% VC, 1.5% TEOA, and 0.1 mM Eosin Y at 120 s light exposure were used to form the hydrogels). Average pore sizes of (**c**) GelMA/Bio-IL and (**d**) PEGDA/Bio-IL hydrogels at varying polymer concentrations and polymer/Bio-IL ratios. Swelling ratios of (**e**) GelMA/Bio-IL and (**f**) PEGDA/Bio-IL hydrogels at 15% final polymer concentration and 50/50 polymer/Bio-IL ratio in DPBS after 4, 8 and 24 h. Degradation of (**g**) GelMA/Bio-IL and (**h**) PEGDA/Bio-IL hydrogels at 15% final polymer concentration and 50/50 polymer/Bio-IL ratio in DPBS supplemented with 10% FBS over a two-week period. Error bars indicate standard error of the means, asterisks mark significance levels of p < 0.05 (*), p < 0.01 (**), and p < 0.001 (***).

The porosity of the hydrogels is an important factor in the modulation of cell and tissue interactions, as well as in the penetration of cells into the scaffold, in both 2D culture and 3D cell encapsulation^58^. Therefore, we aimed to characterize the porosity of GelMA/Bio-IL (Fig. 4a) and PEGDA/Bio-IL (Fig. 4b) hydrogels, using scanning electron microscopy (SEM). SEM analysis determined that the average size of the pores in the GelMA/Bio-IL (Fig. 4c) and PEGDA/Bio-IL (Fig. 4d) hydrogels is also dependent on the final polymer concentration, as well as the polymer/Bio-IL ratio. For instance, the overall pore sizes of 50/50 GelMA/Bio-IL hydrogels decreased from 85.1 ± 18.4 μm to 61.5 ± 13.1 μm, by increasing the final polymer concentration from 10% to 20%. Similarly, the dimensions of the pores decreased from 86.4 ± 20.3 μm to 39.0 ± 7.9 μm, by increasing the ratio of GelMA to Bio-IL from 100/0 to 20/80 at 15% final polymer concentration. Although, a similar trend could be observed for PEGDA/Bio-IL hydrogels, we were not able to observe any apparent pores in all 20/80 hydrogels, as well as 50/50 hydrogels formed at 15% and 20% final polymer concentrations (Fig. 4d). Previous studies have shown that hydrogels with large pores allow better cell penetration, as well as new tissue formation within the microstructure of the scaffold, when compared to hydrogels with lower porosity^58, 59^. The tunable porosity of Bio-IL conjugated hydrogels can be used to modulate the spatial distribution of cells within the scaffolds.

In the context of tissue engineering, excessive water uptake and degradation could potentially lead to impaired mechanical and electroconductive properties. Therefore, we evaluated the water uptake ability and the *in vitro* degradation rate of the composite hydrogels. These results demonstrated that the maximum swellability was achieved after 4 h of incubation, with no further increases observed after 8 and 24 h. In addition, the swelling ratio after 4 h of incubation for 15% GelMA/Bio-IL (Fig. 4e) and PEGDA/Bio-IL (Fig. 4f) hydrogels, increased from 38.1% ± 5.0% to 83.3% ± 8.6%, and from 11.6% ± 3.6% to 185.7% ± 17.1%, respectively, by changing the polymer/Bio-IL ratio from 100/0 to 20/80. In general, the highest swelling ratios for GelMABio-IL (Fig. 4e) and PEGDA/Bio-IL (Fig. 4f) hydrogels were observed in hydrogels synthesized using a polymer/Bio-IL ratio of 20/80. These observations can be explained in part due to the presence of hydroxyl (-OH) and amine (-NH_2_) hydrophilic residues in the choline acrylate structure, which would lead to increased water uptake into the scaffold^60^. Previous studies have reported that the swellability of IL-based hydrogels is highly dependent on the concentration of IL in the system^61, 62^. In our experiments, the highest swelling ratio observed (223.7 ± 28.5) was lower than that reported for other IL conjugated polymers, such as chitosan (>400%)^63^. This characteristic is remarkably advantageous for the implementation of Bio-IL functionalized hydrogels in the context of physiological wet tissues.

Lastly, we aimed to evaluate the *in vitro* degradation of the engineered composite hydrogels. For this, we incubated Bio-IL conjugated hydrogels in Dulbecco’s phosphate-buffered saline (DPBS) and DPBS with 10% fetal bovine serum (FBS), at 37 °C for 14 days. Our results showed that *in vitro* degradation of 15% GelMA/Bio-IL (Fig. 4g) and PEGDA/Bio-IL (Fig. 4h) hydrogels occurred mainly after 24 h of incubation for all polymer/Bio-IL ratios tested. Furthermore, the results also showed that the rate of degradation decreased consistently, by increasing the ratio of polymer to Bio-IL. After 14 days of incubation in DPBS supplemented with 10% FBS, 52.70 ± 4.24 % of 20/80 PEGDA/Bio-IL hydrogel degraded, which was considerably higher than 36.59 ± 3.95 % of 20/80 GelMA/Bio-IL hydrogel. This general trend was also observed for 10%, and 20% GelMA/Bio-IL (Figure S5) and PEGDA/Bio-IL (Figure S6) hydrogels at varying polymer/Bio-IL ratios, both in DPBS and DPBS supplemented with 10% FBS. In general, composite hydrogels containing lower concentrations of Bio-IL exhibited slower degradation rates after 1, 7 and 14 days of incubation. It is important to note that the due to the organic nature of this compound, the degradation of the Bio-IL functionalized hydrogels will not result in the generation of any cytotoxic byproducts^35^.

Taken together, the physical characterization of Bio-IL conjugated hydrogels demonstrated that the microarchitecture, electrical conductivity, porosity, and *in vitro* degradation can be modulated by varying the final polymer concentration, as well as the polymer/Bio-IL ratio. This remarkable degree of tunability suggests that Bio-IL conjugated hydrogels could be readily tailored to different biomedical and tissue engineering applications.

## ***In vitro*** 2D cell seeding on the engineered GelMA/Bio-IL hydrogels

Hydrogels with electroconductive properties possess a remarkable potential for tissue engineering, since they can serve as bioactive scaffolds to modulate excitable cell types. In particular, the use of various synthetic and natural biomaterials has been investigated for the engineering of functional tissue constructs for different tissue engineering applications^64, 65^. Naturally derived polymers, such as collagen and its denatured form gelatin, have been shown to efficiently promote the attachment, proliferation, and migration of various cell types *in vitro* ^2, 66^. This is mainly due to the presence of cell-binding motifs and protease-sensitive degradation sites that modulate the attachment of cells to the ECM *in vivo*. In contrast, synthetic materials such as PEG and its acrylated form, PEGDA, exhibit no intrinsic cell-binding activity due to inert nature of PEG-based polymers^67^. Therefore, we aimed to investigate the potential of GelMA/Bio-IL conjugated hydrogels to support the growth, spreading and function of primary rat CMs in 2D cultures *in vitro*. A commercial live/dead assay was used to determine the viability of CMs growing on the surface of GelMA (Figure S7a) and GelMA/Bio-IL (Figure S7b) hydrogels, over a period of 5 days. Similarly, cell attachment and spreading on GelMA (Figure S7c) and GelMA/Bio-IL (Figure S7d) hydrogels were evaluated through F-actin/DAPI immunofluorescent staining. We also investigated the ability of GelMA/Bio-IL scaffolds to maintain the native phenotype and function of primary CMs in 2D cultures *in vitro*. For this, we evaluated the expression of the cardiac differentiation marker sarcomeric α-actinin (Figures S7h and i), as well as the contractile behavior (Figure S7j) of primary CMs seeded on the surface of 15% (w/v) GelMA and 50/50 GelMA/Bio-IL hydrogels. Our results demonstrated that the viability of CMs seeded on engineered hydrogels was not affected due to the presence of the Bio-IL (Figure S7e). Cells seeded on the surface of GelMA/Bio-IL hydrogels appeared to exhibit slightly lower viabilities at day one post-seeding, when compared to GelMA controls. However, there were no statistically significant differences between the composite scaffolds and the controls, at days 3 and 5 post-seeding. Furthermore, the metabolic activity of the primary cultures was shown to increase consistently throughout the duration of the culture, as shown by the commercial PrestoBlue assay (Figure S7f). This behavior could be explained in part due to the adaptation of the CMs to the conditions *in vitro*, as well as the proliferation of CFs that could not be removed during CM isolation. In addition, GelMA/Bio-IL hydrogels contained significantly higher cell numbers than pure GelMA controls at day 5 post-seeding, as shown by quantification of DAPI-stained cell nuclei (Figure S7g).

CMs maintained in 2D environments tend to revert to a less mature phenotype and lose the ability to respond to physiological stimuli^68^. Thus, apart from the maintenance of metabolically active cells, the preservation of the native phenotype is critical to promote the spatial and functional organization of CMs^69^. Immunofluorescent staining revealed that primary CMs on the surface of GelMA/Bio-IL hydrogels exhibited homogeneous distribution of sarcomeric α-actinin (Figure S7i), compared to the intermittent pattern observed in GelMA controls (Figure S7h). Moreover, these images also showed that CMs were arranged in spatially-relevant multi-cellular organizations and exhibited characteristic cross-striations after 7 days of culture, which are indicative of the sarcomeric structures present in the native ventricular myocardium. In addition, CMs growing in GelMA/Bio-IL hydrogels exhibited a more robust and stable spontaneous contraction profile, when compared to CMs growing on GelMA controls (Figure S7j). For example, on day 7, the beating frequency of CMs seeded on GelMA/Bio-IL was 83.05 ± 9.54 beats/min, which was significantly higher than the beating frequency of cells seeded on pure GelMA hydrogels (52.61 ± 5.43 beats/min). These results suggested that GelMA/Bio-IL hydrogels promoted the formation of interconnected cellular networks on the surface of the scaffolds, which in turn aided to maintain tissue-level function of primary CMs *in vitro*.

Although PEGDA/Bio-IL hydrogels could not be used for cell culture due to the intrinsic inert nature of PEGDA, previous groups have reported the modification of PEG-based hydrogels via tethering of bioactive motifs, such as the incorporation of Arg-Gly-Asp (RGD) and matrix metalloproteinase-sensitive degradation domains^70–72^. These modifications have been shown to enhance the cell-binding ability and the biodegradability of PEG-based hydrogels for tissue engineering applications. Furthermore, due to their intrinsic resistance to cell adhesion and protein adsorption, PEG-based hydrogels exhibit minimal adverse host responses and highly specific bioactivity via the conjugation of specific bioactive agents^73^. The intrinsic differences between PEGDA and GelMA polymers clearly demonstrate the remarkable potential of Bio-IL functionalization to engineer new conductive biomaterials with a wide range of biological and physicochemical properties, which can be tailored for different biomedical applications.

## 3D encapsulation of primary CMs/CFs inside the engineered GelMA/Bio-IL hydrogels

**Figure 5:**
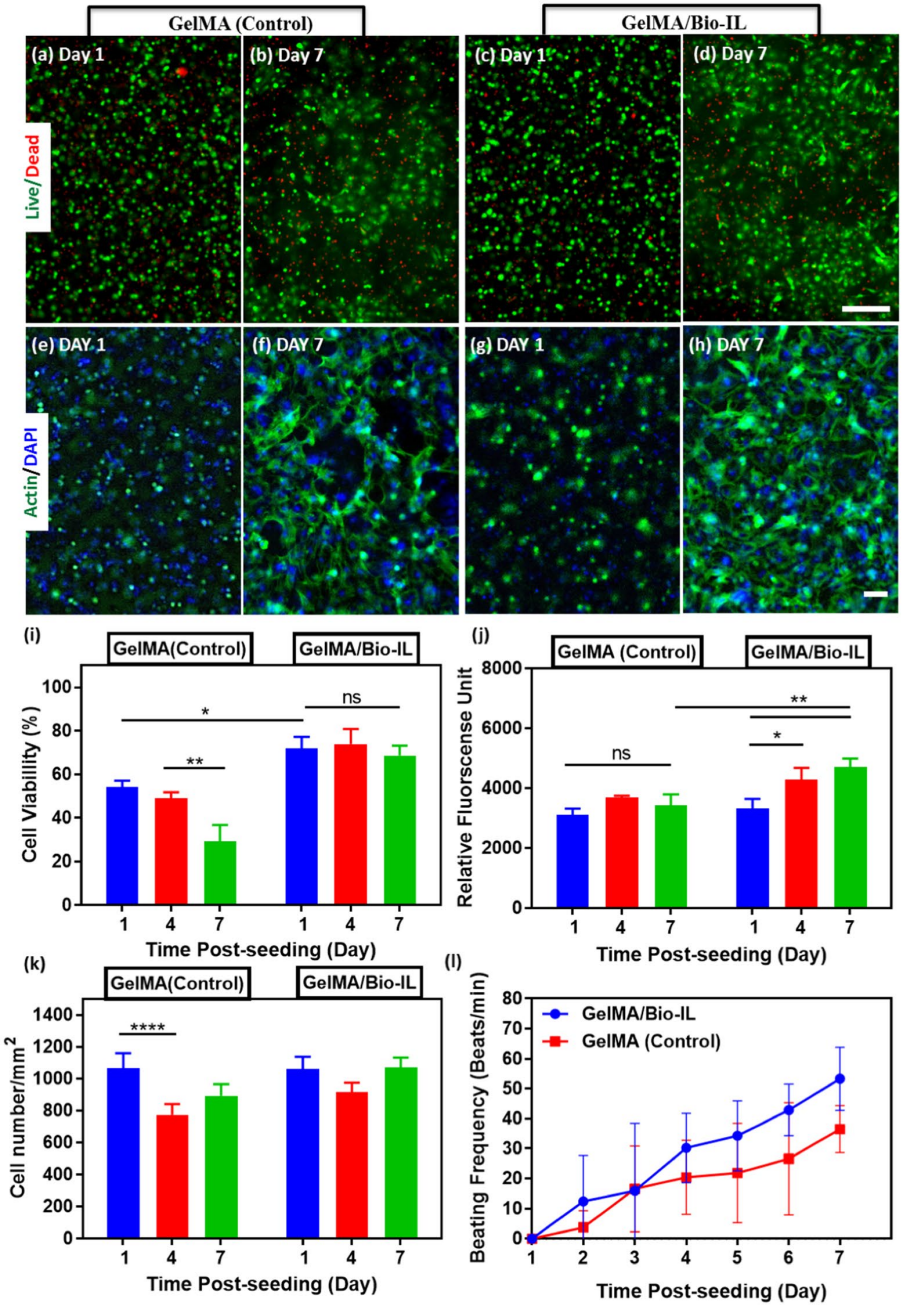
*In vitro* 3D encapsulation of cardiomyocytes (CMs) and cardiac fibroblasts (CFs) in GelMA/Bio-IL hydrogels. CMs and CFs (2:1 ratio) were 3D encapsulated inside visible light-crosslinked GelMA (control) and GelMA/Bio-IL hydrogels. Representative live/dead images from CMs/CFs encapsulated in GelMA **(a**,**b)** and GelMA/Bio-IL **(c**,**d)** hydrogels at days 1 and 7 post-encapsulation. Representative F-Actin/DAPI fluorescent images of CMs/CFs encapsulated in GelMA **(e**,**f)** and GelMA/Bio-IL **(g**,**h)** hydrogels, at days 1 and 7 post-encapsulation (scale bar = 200 μm). **(i)** Quantification of cell viability for 3D encapsulated CMs/CFs at days 1, 4, and 7 post-encapsulation. **(j)** Quantification of metabolic activity, relative fluorescence units (RFU), using PrestoBlue assay at days 1, 4, and 7 post-encapsulation. **(k)** Quantification of cell proliferation based on DAPI-stained cell nuclei at days 1, 4, and 7 post-encapsulation. **(l)** Characterization of synchronous contraction of CMs/CFs encapsulated in GelMA (control) and GelMA/Bio-IL hydrogels over 7 days of culture. (*p < 0.05, **p < 0.01, ***p < 0.001 and ****p < 0.0001). All hydrogels were synthesized using 15% (w/v) final polymer concentration and 50/50 GelMA/Bio-IL ratio. Error bars indicate standard error of the means, asterisks mark significance levels of p < 0.05 (*)p < 0.01 (**), and p < 0.001 (***).

Biomaterial-based cardiac tissue models that rely on 2D cultures of primary CMs have been extensively reported in the literature to investigate many cellular and biochemical mechanisms relevant for cardiac physiology^64^. More recently, *in vitro* models with increased physiological relevance have been engineered to recapitulate the interactions between different cell types and the 3D architecture of native tissues^74^. However, encapsulation of primary cardiac cells in highly biocompatible ECHs with tunable electroconductive and mechanical properties has not been fully explored. Here, we investigated the ability of GelMA/Bio-IL hydrogels to support the growth and function of 3D encapsulated CMs and CFs *in vitro*. Similar to 2D cultures, we used a live/dead assay, F-actin/DAPI staining, and a PrestoBlue assay to determine the viability (Fig. 5a–d), spreading (Fig. 5e–h), and metabolic activity (Fig. 5j) of 3D encapsulated cells in 15% (w/v) 50/50 GelMA/Bio-IL hydrogels, respectively. Although the overall viability of 3D encapsulated cells was comparatively lower than that observed for 2D cultures (Figure S7e), cells in GelMA/Bio-IL hydrogels exhibited significantly higher viabilities as compared to pure GelMA hydrogels (Fig. 5i). In addition, our results also showed that the metabolic activity of cells encapsulated in GelMA/Bio-IL hydrogels increased consistently during the 5 days of culture (Fig. 5j). However, no significant differences between GelMA/Bio-IL hydrogels and GelMA controls were observed regarding the proliferation of 3D encapsulated cells (Fig. 5k). Lastly, similar to what we observed for 2D cultures, our results demonstrated that CMs encapsulated in GelMA/Bio-IL hydrogels exhibited comparatively better contractile profiles as compared to pure GelMA controls (Fig. 5l). Overall, *in vitro* assessment of GelMA/Bio-IL hydrogels demonstrated that the engineered ECHs are cytocompatible and promote cell growth and spreading in 3D cultures *in vitro*. Although no statistically significant differences in cell numbers could be observed (Fig. 5k), our results showed that the viability (Fig. 5i) and metabolic activity (Fig. 5j) of CMs/CFs 3D encapsulated in GelMA/Bio-IL hydrogels were higher, when compared to pure GelMA controls. The mechanism underlying the improved growth of 3D encapsulated CMs in GelMA/Bio-IL hydrogels was not investigated in this study. However, previous studies have described that electrical stimuli could influence cell fate, through the modulation of the calcium/calmodulin pathway^75, 76^. Briefly, electrical stimuli propagate across the cell membrane, raising the intracellular calcium concentration by activating voltage-gated calcium channels. Elevated intracellular calcium levels activate the cytoskeletal form of calmodulin, which results in enhanced proliferation and increased expression of vascular endothelial growth factor (VEGF), and transforming growth factor (TGF)-ß^77^. This characteristic of GelMA/Bio-IL hydrogels could be critical in the restoration of impaired electrical conductivity and tissue function in scarred peri-infarct regions.

*In vitro* 3D co-cultures of CMs and CFs have been explored to engineer myocardial tissue constructs for drug screening and tissue engineering applications^78–80^. However, the successful 3D encapsulation of CMs/CFs in highly biocompatible hydrogels with tunable mechanical and electroconductive properties has not been fully explored. Apart from material biocompatibility, the integration of physiological stimuli is critical to promote growth, survival, and the functional organization of excitable cell types, such as nerve and muscle^69, 81^. After myocardial infarction, the nonconductive nature of the resulting scar tissue leads to ventricular dysfunction, as well as the electrical uncoupling of viable CMs in the infarcted region^82^. Due to the limited regenerative potential of adult CMs, several regenerative cardiac tissue engineering approaches have been developed using two main strategies: cell-based and/or material-based scaffolds^83^. However, one of the major limitations of conventional biomaterial-based approaches is that the insulating polymeric scaffolds diminish the transfer of electrical signals between CMs, which could lead to arrhythmias after implantation^84^. Thus, ECHs could be used to engineer scaffolds that promote impulse propagation and synchronize contraction, which in turn could help restore ventricular function by electrically coupling isolated CMs to the native tissue^53^.

## ***In vivo*** biodegradation and biocompatibility of GelMA/Bio-IL hydrogels

**Figure 6:**
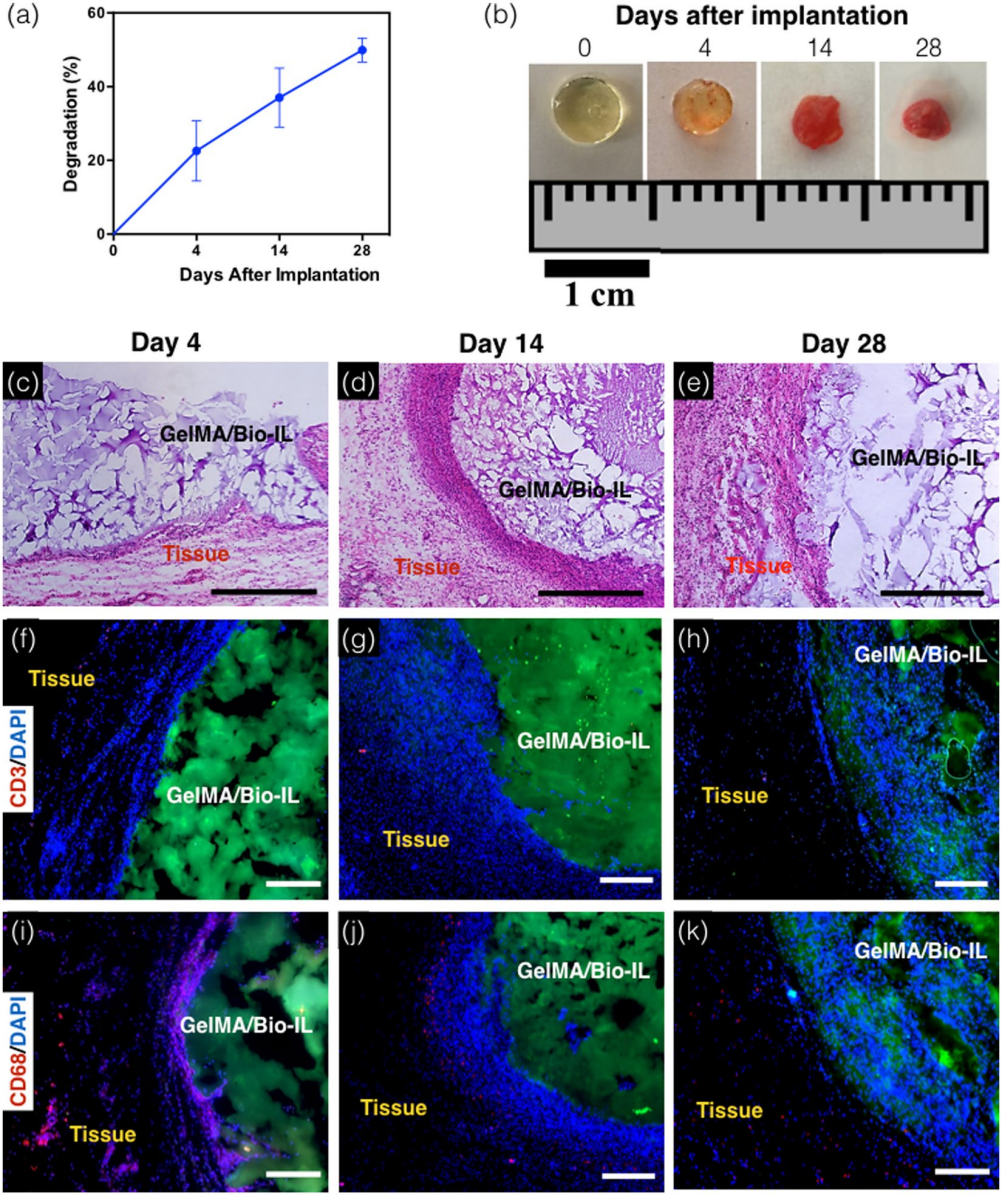
*In vivo* biodegradation and biocompatibility of GelMA/Bio-IL composite hydrogels using a rat subcutaneous model. (**a**,**b**) Evaluation of the *in vivo* degradation of GelMA/Bio-IL on days 0, 4, 14 and 28 post implantation (n = 4). (**a**) *In vivo* degradation of GelMA/Bio-IL hydrogels based on weight loss of the implants. The *in vivo* degradation profile of GelMA/Bio-IL hydrogels exhibited an approximately linear behavior throughout 28 days after implantation. (**b**) Images of the GelMA/Bio-IL composite hydrogels at 0, 4, 14, and 28 days after implantation. (**c**–**e**) Hematoxylin and eosin (H&E) staining of GelMA/Bio-IL sections after (**c**) 4, (**d**) 14, and (**e**) 28 days of implantation (scale bars = 500 µm). The H&E images shows a negligible amount of inflammatory cells. (**f**–**h**) Fluorescent immunohistochemical analysis of subcutaneously implanted GelMA/Bio-IL hydrogels showing no significant local lymphocyte infiltration (CD3) at days (**f**) 4, (**g**) 14 and (**h**) 28 (scale bars = 200 µm), and significant macrophage (CD68) presence at day (**i**) 4, which disappeared at days (**j**) 14 and (**k**) 28 (scale bars = 200 µm). Green, red and blue colors in (**f**–**k**) represent the GelMA/Bio-IL hydrogels, the immune cells, and the cell nuclei (DAPI). 1% VC, 1.5% TEOA, and 0.1 mM Eosin Y at 120 s light exposure were used to form the hydrogels. All hydrogels were synthesized using 15% final polymer concentration and 50/50 GelMA/Bio-IL ratio.

One of the limitations of conventional conductive polymers is that they are often not biodegradable *in vivo*, which could trigger a persistent inflammatory response due to their prolonged half-lives in the organism^19^. We investigated the *in vivo* degradation and interactions of GelMA-Bio-IL hydrogels with the local tissues, as well as their immunogenicity profile when implanted subcutaneously *in vivo* in an animal host. Explanted samples, recovered at days 4, 14, and 28 post-implantation, revealed that GelMA/Bio-IL hydrogels exhibited sustained biodegradation throughout the duration of the 28-day experiment (Fig. 6a). This observation suggested that the engineered hydrogels were efficiently degraded *in vivo*, through enzymatic hydrolysis of the hydrogel matrix. Visual inspection of the explanted samples also revealed significant infiltration of host tissues within the hydrogels (Fig. 6b). Although complete biodegradation of the scaffold was not observed due to the length of the study, previous works have demonstrated that GelMA-based hydrogels contain peptide sequences that facilitate cell-mediated degradation *in vivo* ^85–87^. Furthermore, the conjugation of the Bio-IL to different polymers could be used to engineer ECHs with varying degrees of biodegradability. For example, biomaterials with more hydrolytically-stable backbones such as PEGDA would result ideal for long-term applications, where a more biostable implantable scaffold is needed^88^.

One crucial characteristic of implantable hydrogel-based scaffolds for tissue engineering is their ability to be efficiently biodegraded, in a period of time that allows the growth of new autologous tissue. Since both the mechanical and electroconductive properties of Bio-IL functionalized hydrogels rely on their physical integrity, sustained biodegradation will certainly influence the performance of the scaffolds *in vivo*. However, our results demonstrated that the degradation rate of Bio-IL hydrogels can be readily tuned by varying the final polymer concentration and the polymer/Bio-IL ratio (Fig. 4g and h). As opposed to conventional methods used to engineer ECHs, Bio-IL functionalization provides homogeneous and intrinsic electroconductive properties to the entire polymer network. This in turn suggests that the decay in the mechanical and electroconductive performance of the engineered hydrogels is directly proportional to the degradation rate. Therefore, the biodegradability of Bio-IL functionalized hydrogels could be further optimized depending on the specific application, to ensure adequate mechanical and electroconductive performance during the desired period of time.

The biodegradability profile of GelMA/Bio-IL hydrogels allows for sustained cellular ingrowth, as well as the eventual replacement of the implanted sample with new autologous tissue. Accordingly, histological assessment of the explanted hydrogels revealed ingrowth of predominantly non-inflammatory tissue, as well as low deposition of a fibrous collagenous capsule (Fig. 6c–e). This observation was further confirmed by immunohistofluorescent analysis of the explanted samples. Fluorescent immunostaining of the CD3 antigen revealed no sustained infiltration of pro-inflammatory leukocytes (Fig. 6f–h). In addition, implantation of GelMA/Bio-IL hydrogels elicited a macrophage response by expression of CD68 antigen, initially at day 4; however, they were significantly reduced by day 28 (Fig. 6i–k). These results demonstrated that GelMA/Bio-IL hydrogels elicited minimal inflammatory responses *in vivo*. In addition, specific physiological responses could be triggered through the use of different biopolymers. For example, previous studies have demonstrated the remarkable suitability of GelMA-based hydrogels for the induction of angiogenesis^89^. In contrast, the conjugation of Bio-IL to the bioactive polymer alginate could be used for studies involving osteogenesis, as well as other bone tissue engineering applications^90^.

The physicochemical cues from the extracellular microenvironment play a key role in various physiological and pathological processes that modulate tissue function^91^. For example, after myocardial infarction, the nonconductive nature of the resulting scar tissue leads to the electrical uncoupling of the infarcted area, and eventually to ventricular dysfunction^82^. Due to the limited regenerative potential of adult CMs, several biomaterials-based tissue engineering approaches for myocardium regeneration have been developed^92^. However, the non-conductive nature of most biopolymers greatly diminishes the propagation of electrical stimuli across the scaffold^84^. Thus, biomaterials-based approaches, like the one presented in this work, could help restore ventricular function by mechanically and electrically coupling the area around the infarcted myocardium^53^. Future work will investigate mechanical and electrical recoupling of impaired myocardium using GelMA/Bio-IL composite hydrogels. Furthermore, in addition to its role in the excitation-contraction coupling, electrical stimulation of CMs is also known to modulate cell proliferation and function^93, 94^. Therefore, the ability of GelMA/Bio-IL composite hydrogels to efficiently transduce multiple physiological stimuli to modulate tissue function, holds a remarkable potential for cardiac tissue engineering applications.

## Conclusion

We introduced a new method to generate electroconductive polymer-based biomaterials through the conjugation of a choline-based Bio-IL to different polymers. This approach can be used to engineer new conductive biomaterials from inherently non-conductive polymers with different biochemical and biophysical properties. We described the conjugation of a Bio-IL to the two widely used polymers GelMA and PEGDA. We demonstrated that the mechanical properties, coductivity, porosity, degradability, and swellability of the engineered hydrogels could be finely-tuned by varying the final polymer concentration, as well as the ratio of polymer to Bio-IL. We also investigated the ability of Bio-IL conjugated hydrogels to transduce physicochemical stimuli, and modulate the growth of primary CMs in both 2D and 3D cultures *in vitro*. We demonstrated that the engineered hydrogels were highly biodegradable and biocompatible *in vitro*, and that they did not elicit inflammatory responses when implanted *in vivo*. Taken together, our results demonstrated that Bio-IL conjugated hydrogels could be implemented and readily tailored to different biomedical and tissue engineering applications.

## Experimental Section

### Synthesis of conductive hydrogels

ECHs were synthesized by mixing different ratios of methacrylated polymers and choline acrylate (Bio-IL). Bio-IL was synthesized by mixing choline bicarbonate with acrylic acid at a 1:1 mole ratio. The mixture was reacted at 50 °C for 5 h, then purified by evaporation overnight in a vacuum at room temperature. GelMA and PEGDA were the two polymers used to form ECHs with Bio-IL. GelMA was synthesized using a method previously described in the literature^45^. Briefly, 10% (w/v) gelatin solution was reacted with 8 mL of methacrylic anhydride for 3 h. The solution was then dialyzed for 5 days to remove any unreacted methacrylic anhydride, and then placed in a −80 °C freezer for 24 h. The frozen acrylated polymer was then freeze-dried for 7 days. The PEGDA used for this research was purchased from Sigma Aldrich (average M_n_ 700). To form polymer/Bio-IL hydrogels, the prepolymer and Bio-IL were added to distilled water at varying final polymer concentrations and polymer/Bio-IL ratios, then mixed with 1.5% TEOA, 1% VC, and 0.1 mM Eosin Y, which acted as the photoinitiators. Hydrogels were then rapidly photocrosslinked in the presence of visible light at a wavelength of 475 nm for 120 s, using a LS1000 FocalSeal Xenon Light Source, manufactured by Genzyme.

### ^1^H NMR characterization of GelMA/Bio-IL hydrogels

^1^H NMR analyses were performed to characterize GelMA/Bio-IL composite hydrogels using a Varian Inova-500 NMR spectrometer. ^1^H-NMR spectra were obtained from choline bicarbonate, choline acrylate (Bio-IL), GelMA prepolymer, and GelMA/Bio-IL hydrogels. Methacrylated groups were identified due to the presence of peak values at δ = 5.3 and 5.7 ppm. The decreasing rate for the C=C double bond signals $(-\frac{\partial (C=C)}{\partial t})$ in methacrylate group of GelMA was associated with the extent of crosslinking of composite hydrogel as well as conjugation of Bio-IL to GelMA. This area decrease was calculated using the following equation:

1 $${\bf{D}}{\bf{e}}{\bf{c}}{\bf{a}}{\bf{y}}\,{\bf{o}}{\bf{f}}\,{\bf{m}}{\bf{e}}{\bf{t}}{\bf{h}}{\bf{a}}{\bf{c}}{\bf{r}}{\bf{y}}{\bf{l}}{\bf{a}}{\bf{t}}{\bf{e}}\,{\bf{g}}{\bf{r}}{\bf{o}}{\bf{u}}{\bf{p}}\,( \% )=(\frac{{\bf{P}}{{\bf{A}}}_{{\bf{b}}}-{\bf{P}}{{\bf{A}}}_{{\bf{a}}}}{{\bf{P}}{{\bf{A}}}_{{\bf{b}}}})\times {\bf{100}}$$

where ***PA***_***b***_ and ***PA***_***a***_ represent the peak areas of methacrylated groups before and after photocrosslinking, respectively. Accordingly, ***PA***_***b***_ − ***PA***_***a***_ corresponds to the concentration of methacrylated groups consumed in the photocrosslinking process. ACD/Spectrus NMR analysis software were used to integrate the area under the peaks and all the data was analyzed with respect to phenyl group peaks at δ = 6.5–7.5 ppm.

### Mechanical testing

Mechanical testing on engineered ECHs were performed by using an Instron 5542 mechanical tester. Both the elastic and compressive modulus were analyzed for each of the hydrogels with varying final polymer concentration and polymer/Bio-IL ratios. Composite hydrogels were created by using PDMS molds, which formed rectangular hydrogels (length: 12.00 mm, width: 5.00 mm, depth: 1.25 mm) for tensile tests and cylindrical hydrogels (diameter: 5.5 mm, height: 4 mm) for compression tests. A volume of 70 *μ*L of prepolymer solution was used to form photocrosslinked ECHs. ECHs were allowed to swell in DPBS for 4 h at 37 °C prior to mechanical testing. At least 5 samples were tested for each condition.

For the compression tests, hydrogels were placed between two compression plates, and compression was applied to each sample at a rate of 1 mm/min. Compression (mm) and load (N) were recorded during each test using Bluehill software. Compression modulus was calculated as the tangent slope of the initial linear region of the stress-strain curve between 0 mm/mm and 0.1 mm/mm compressive strain. For the tensile tests, hydrogels were held between two tensile grips and stretched at a rate of 1 mm/min until failure. The elastic modulus was calculated as the tangent slope of the stress-strain curve. At least 5 samples were tested per condition to obtain average and standard deviation.

### *In vitro* evaluation of electrical conductivity

ECHs with varying final polymer concentration and polymer/Bio-IL ratios were crosslinked in a rectangular PDMS mold and allowed to dry for 24 h. Once dried, conductivity measurement was performed using a two-probe electrical station connected to a Hewlett Packard 4155A Semiconductor Parameter analyzer. The dimensions of each hydrogel were measured and placed in a relaxed state where the two probes penetrated the hydrogels - one at each end (Fig. 2a). The HP 4155A was set to measure current in the presence of an electrical stimulation ranging from −25 to 25 volts. The results were then analyzed to determine conductivity values. At least 5 samples were tested for each condition.

### *Ex vivo* evaluation of electrical conductivity

Adult female Wistar rats were provided by the Institutional Animal Care and Use Committee (ICAUC) at Northeastern University (Boston, MA, USA). All experiments were performed in accordance with relevant guidelines and regulations. Immediately after euthanasia, the rectus abdominis muscles were dissected and cut into 10 × 10 mm pieces. The tissue samples were then transferred to a glass petri dish, and placed 3 mm apart on the opposite sides of an insulating PDMS mold (Fig. 2e). GelMA/Bio-IL hydrogels at 50/50 and 20/80 ratios, as well as pure GelMA controls were photocrosslinked *in situ* to connect the two pieces of tissue. 50 ms square pulses of direct current were applied to the tissue using an Agilent wave generator (Agilent 33220A). The electrical stimulation was applied to the samples using short platinum wires with 0.25 mm diameter and 99.9% trace metal basis, bought from Sigma-Aldrich (MO, USA). The threshold at which contraction was achieved was determined by applying increasing voltages (1–10 V) at a constant frequency of 1 hertz. Muscle contraction was visually inspected at each voltage, after applying electrical stimulation to the sample on the opposite side of the mold.

### *In vitro* degradation test

ECHs were fabricated as previously explained for compression test. ECHs were then freeze-dried, weighed and were placed in 24-well plate with 1 ml of DPBS or DPBS supplemented with 10% FBS solutions at 37 °C in an oven continuously for 2 weeks. The DPBS/FBS solutions were refreshed every 3 days to maintain constant enzyme activity. At prearranged time points (after 1, 7 and 14 days), the DPBS/FBS solutions were removed and the samples were freeze-dried for 24 h and weighed. The percentage degradation (D%) of the hydrogels was calculated using the below equation:

2 $${\boldsymbol{D}}\, \% =\frac{{{\boldsymbol{W}}}_{{\boldsymbol{i}}}-{{\boldsymbol{W}}}_{{\boldsymbol{t}}}}{{{\boldsymbol{W}}}_{{\boldsymbol{i}}}}\times {\bf{100}} \% ,$$

where W_i_ is the initial dry weight of the sample and W_t_ is the dry weight after time t.

### Swelling ratio measurements

The equilibrium swelling ratio of GelMA/Bio-IL and PEGDA/Bio-IL composite hydrogels were evaluated. For this purpose, cylinder-shaped hydrogels were prepared (7 mm in diameter, 2 mm in depth) as described previously. Prepared hydrogels were washed three times with DPBS. Then, they were lyophilized and weighed in dry conditions. Thereafter, the samples were immersed in DPBS at 37 °C for 4, 8 and 24 h and weighed again after immersion. The swelling ratio and water uptake capacity of the samples were calculated as the ratio of the swelled sample mass to the mass of lyophilized sample.

### SEM analysis

SEM analysis was performed to evaluate the porosity of the engineered GelMA/Bio-IL and PEGDA/Bio-IL hydrogels. The samples were prepared in a similar procedure as described for swelling ratio tests. The freeze-dried samples were then coated by gold/palladium (Au/Pd) before SEM analysis. The SEM images were acquired by using a Hitachi S-4800 SEM (10 kV). Pore size analysis of the GelMA/Bio-IL hydrogels was performed by measuring the pore sizes of at least three images of four samples (n = 50) using ImageJ software.

### Primary CM isolation

Primary rat CMs were isolated from 2-day-old neonatal Sprague Dawley pups according to the protocol approved by the ICAUC at Northeastern University. All experiments were performed in accordance with relevant guidelines and regulations. Briefly, pups were quickly decapitated with scissors after disinfecting the neck and sternum with 70% ethanol. A vertical incision was made across the sternum to excise the heart, which was placed in cold Hank’s Balanced Salt Solution (HBSS) buffer. The atria and blood vessels were carefully removed and each heart was quartered and incubated overnight in a solution of 0.05% (w/v) trypsin in HBSS at 4 °C. Trypsin digestion was stopped by adding culture media, followed by shaking for 5 min at 37 °C in a water bath. The tissues were then serially digested in 0.1% collagenase type II solution in HBSS (10 min shaking incubation at 37 °C). The collagenase solution with the CMs were centrifuged at 500 × g for 5 min. Primary cells were resuspended in DMEM supplemented with 10% FBS and pre-plated for 1 h to enrich for cardiomyocytes.

### Surface seeding (2D culture)

Hydrogels were formed by placing a 7-μl drop of hydrogel precursor in a spacer with 150-μm height and covered by a glass slide coated with 3-(trimethoxysilyl) propyl methacrylate (TMSPMA, Sigma-Aldrich). Hydrogel precursors were then photocrosslinked for 20 s using a Genzyme FocalSeal LS100 xenon light source. Primary rat CMs (3.5 × 10^4^ cells/scaffold) were seeded on the surface of the hydrogels and placed in 24-well plates with 400 μl of growth medium (DMEM supplemented with 10% fetal bovine serum (FBS, Invitrogen) and 1% penicillin/streptomycin (Invitrogen)). 2D cultures were maintained at 37 °C in a 5% CO_2_ humidified atmosphere, for 10 days and culture medium was replaced every 48 h.

### 3D cell encapsulation

Hydrogel precursors were prepared in cell culture medium containing 1.5% TEOA, 1% VC, and 0.1 mM Eosin Y. A cell suspension containing freshly isolated CMs and CFs in culture medium (2:1 ratio of CMs:CFs and 1.8 × 10^7^ cells/ml total cell density) was gently mixed with an equal volume of the precursor solution. 7-µl drops were then pipetted on 150-µm thick spacers, and covered by TMSPMA-coated glass slides. Hydrogels were then photocrosslinked for 120 s using a Genzyme FocalSeal LS100 xenon light source, as described before for 2D cultures. Cell laden hydrogels were placed in 24-well plates with 400 μl of growth medium, and maintained at 37 °C in a 5% CO_2_ humidified atmosphere for 10 days.

### Cell viability

The viability of primary CMs grown on the surface of GelMA and GelMA/Bio-IL hydrogels was evaluated using a commercial live/dead viability kit (Invitrogen), according to instructions from the manufacturer. Briefly, cells were stained with 0.5 μl/ml of calcein AM and 2 μl/ml of ethidium homodimer-1 (EthD-1) in DPBS for 15 min at 37 °C. Fluorescent image acquisition was carried out at days 1, 4, and 7 post-seeding using an AxioObserver Z1 inverted microscope (Zeiss). Viable cells appeared as green and apoptotic/dead cells appeared as red. The number of live and dead cells was quantified using the ImageJ software. Cell viability was determined as the number of live cells divided by the total number of live and dead cells.

### Metabolic activity

The metabolic activity of the cells was evaluated at days 1, 4, 7 post-seeding, using a PrestoBlue assay (Life Technologies) according to instructions from the manufacturer. Briefly, 2D and 3D cultures of primary CMs were incubated in 400 μL of growth medium with 10% PrestoBlue reagent for 2 h at 37 °C. The resulting fluorescence was measured (excitation 530 nm; emission 590 nm) using a Synergy HT fluorescence plate reader (BioTek). Control wells without cells were used to determine the background for all experiments.

### Cell adhesion, proliferation and spreading

CM spreading on the surface of the engineered composite hydrogels was visualized through fluorescent staining of F-actin filaments and cell nuclei. Briefly, 2D cultures at days 1, 4, and 7 post-seeding were fixed in 4% (v/v) paraformaldehyde (Sigma) for 15 min., permeabilized in 0.1% (w/v) Triton X-100 (Sigma) for 5 min, and then blocked in 1% (w/v) bovine serum albumin (BSA, Sigma) for 30 min. Samples were then incubated with Alexa-fluor 488-labeled rhodamine-phalloidin (20/800 dilution in 0.1% BSA, Invitrogen) for 45 min. After three consecutive washes with DPBS, samples were counterstained with 1 μl/ml DAPI (4′,6-diamidino-2-phenylindole, Sigma) in DPBS for 5 min. Fluorescent image acquisition was carried out using an AxioObserver Z1 inverted microscope.

### Immunostaining of cardiac markers

Immunocytofluorescent staining was performed on 2D cultures of primary CMs in order to evaluate the expression of the cardiac differentiation marker sarcomeric α-actinin. Briefly, CMs growing on the surface of GelMA and GelMA/Bio-IL hydrogels gel were fixed in 4% paraformaldehyde for 1 h at room temperature at day 7 post-seeding. Samples were washed three times with DPBS, permeabilized in 0.1% (w/v) Triton X-100 for 30 min, and blocked in 10% (v/v) goat serum in DPBS containing 0.1% Triton x-100 for 1 h. Samples were incubated overnight with anti-sarcomeric α-actinin primary antibody (1:200 dilution) in 10% (v/v) goat serum at 4 °C. After incubation, samples were washed three times with DPBS and incubated for 2 h at room temperature with an Alexa Fluor 488-conjugated secondary antibody diluted in 10% (v/v) goat serum (1:200 dilution). Lastly, the samples were washed three times with DPBS and counterstained with DAPI (1/1000 dilution in DPBS) for 5 min at room temperature. Image acquisition was performed using an AxioObserver Z1 inverted microscope.

### *In vivo* biodegradation and biocompatibility

All animal experiments were reviewed and approved by the ICAUC (protocols 15-0521R and 15-1248R) at Northeastern University, and all experiments were performed in accordance with relevant guidelines and regulations. Male Wistar rats (200–250 grams) were obtained from Charles River (Boston, MA, USA) and housed in the local animal care facility under conditions of circadian day–night rhythm and feeding *ad libitum*. Anesthesia was achieved by 2.0 to 2.5% isoflurane inhalation, followed by 0.02 to 0.05 mg/kg SC buprenorphine administration. After inducing anesthesia, eight 1-cm incisions were made on the posterior medio-dorsal skin, and small lateral subcutaneous pockets were prepared by blunt dissection around the incisions. GelMA/Bio-IL hydrogels (1 × 5 mm disks) were implanted into the pockets, followed by anatomical wound closure and recovery from anesthesia. Animals were euthanized by anesthesia/exsanguination at days 4, 14 and 28 post-implantation, after which the samples were retrieved with the associated tissue and placed in DPBS.

### Histological analysis and immunofluorescent staining

Histological analyses were performed on cryosections of the explanted hydrogel samples in order to characterize the inflammatory response elicited by the implanted material. After explantation, samples were fixed in 4% paraformaldehyde for 4 hours, followed by overnight incubation in 30% sucrose at 4 °C. Samples were then embedded in Optimal Cutting Temperature compound (OCT) and flash frozen in liquid nitrogen. Frozen samples were then sectioned using a Leica Biosystems CM3050 S Research Cryostat. 15-µm cryosections were obtained and mounted in positively charged slides using DPX mountant medium (Sigma). The slides were then processed for hematoxylin and eosin staining (Sigma) according to instructions from the manufacturer. Immunohistofluorescent staining was performed on mounted cryosections as previously reported^29^. Anti-CD3 [SP7] (ab16669) and anti-CD68 (ab125212) (Abcam) were used as primary antibodies, and an Alexa Fluor 594-conjugated secondary antibody (Invitrogen) was used for detection. All sections were counterstained with DAPI (Invitrogen), and visualized on an AxioObserver Z1 inverted microscope (Zeiss).

### Statistical analysis

Data analysis was carried out using a 2-way ANOVA test with the GraphPad Prism 6.0 software. Error bars were calculated as the mean ± standard deviation (SD) of measurements (*p < 0.05, **p < 0.01, and ***p < 0.001).

## Electronic supplementary material

Supporting Information

Supplementary Video 1
