# Supplementary material for: Engineering Biodegradable and Biocompatible Bio-ionic Liquid Conjugated Hydrogels with Tunable Conductivity and Mechanical Properties
Source: Sci Rep. 2017 Jun 28;7:4345. doi: 10.1038/s41598-017-04280-w (PMC5489531; doi:10.1038/s41598-017-04280-w)
Supplement: Supplementary file 1 — Supporting Information [file 41598_2017_4280_MOESM1_ESM.doc]

Supporting Information

**Engineering Biodegradable and Biocompatible Bio-ionic Liquid Conjugated Hydrogels with Tunable Conductivity and Mechanical Properties**

*Iman Noshadi^1,2,3Ψ^, Brian W. Walker^1Ψ^, Roberto Portillo Lara^1,4^, Ehsan Shirzaei Sani^1^, Nayara Gomes^1^,*

*Mohammad Reza Aziziyan^1^, Nasim Annabi^*1,2,3^*


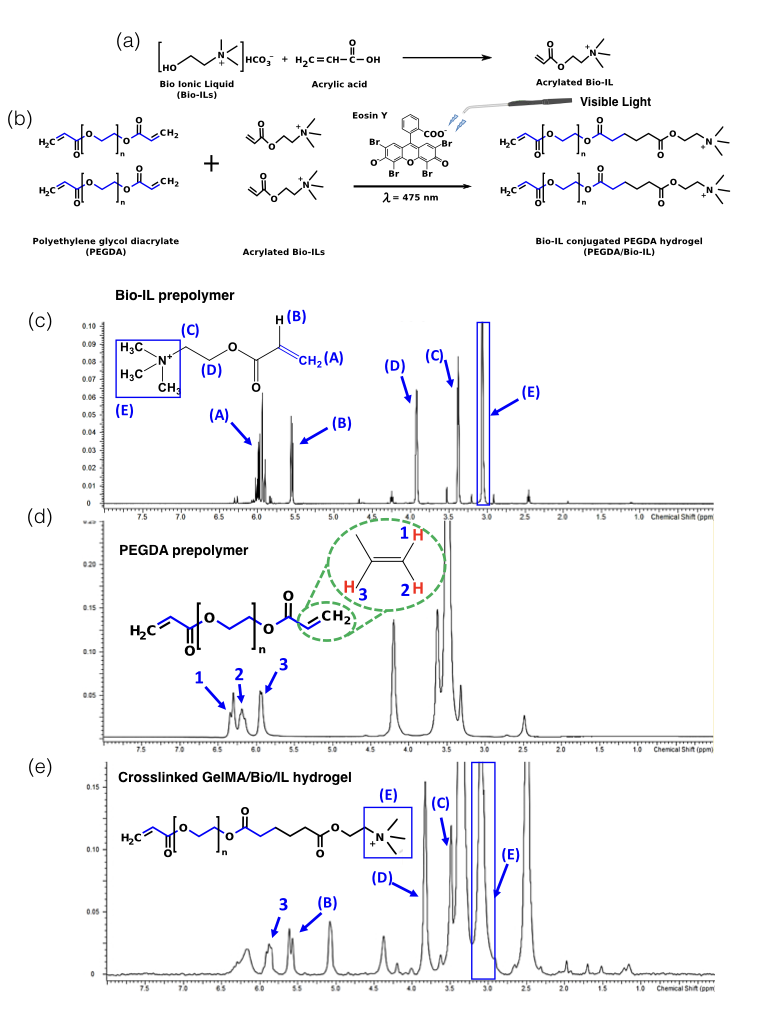


**Figure S1. Synthesis and characterization of Bio-IL functionalized PEGDA hydrogel.** The panels show schematics of the proposed reaction for **(a)** the acrylation of choline bicarbonate to form Bio-IL, and **(b)** the reaction between PEGDA and Bio-IL in the presence of Eosin Y and visible light to form PEGDA/Bio-IL hydrogel. ^1^H-NMR analysis of **(c)** Bio-IL prepolymer, **(d)** PEGDA prepolymer, and **(e)** PEGDA/Bio-IL composite hydrogel. PEGDA/Bio-IL hydrogels were formed by using 1% VC, 1.5% TEOA, and 0.1 mM Eosin Y at 120 s light exposure.


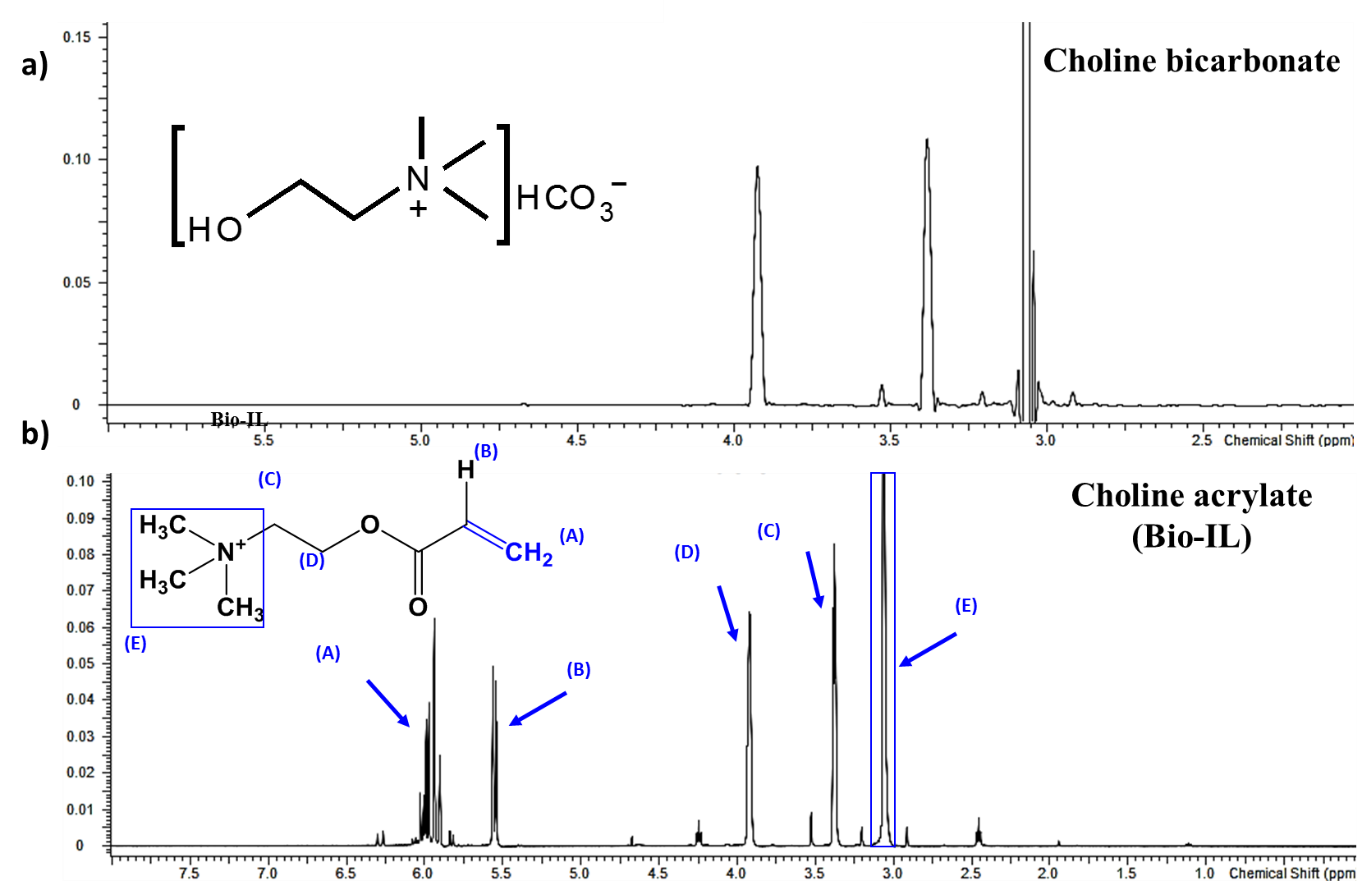


**Figure S2. Characterization of choline bicarbonate and Bio-IL using ^1^H NMR.** NMR spectra of (**a**) choline bicarbonate, and (**b**) choline acrylate (Bio-IL). Bio-IL was synthesized by reacting choline bicarbonate and acrylic acid at 50 °C for 5 h. The presence of peaks at δ=5.8-6.1 ppm confirmed the acrylation of choline bicarbonate (1% VC, 1.5% TEOA, 0.1 mM Eosin Y, and 120 s exposure to visible light were used to form the hydrogels).


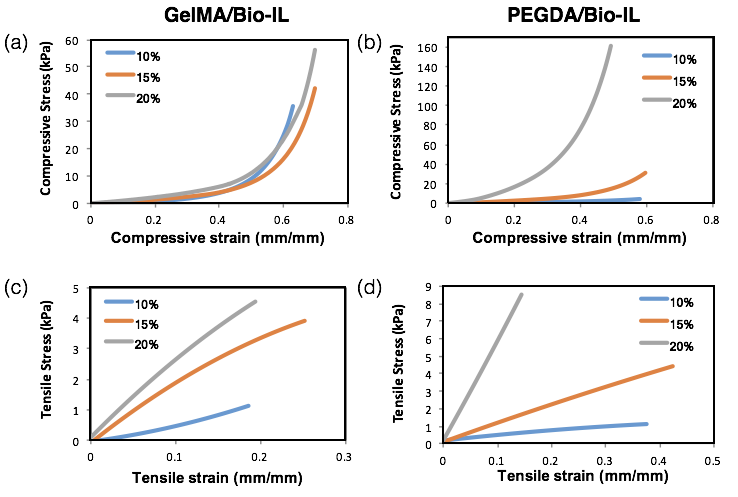


**Figure S3.** **Mechanical properties of Bio-IL functionalized GelMA and PEGDA hydrogel crosslinked with visible light.** 1% VC, 1.5% TEOA, 0.1 mM Eosin Y, and 120 sec exposure to visible light were used to form the hydrogels. Representative compression stress/strain curves for (**a**) GelMA/Bio-IL and (**b**) PEGDA/Bio-IL hydrogels at 80/20 polymer/Bio-IL ratio and various polymer concentrations. Representative tensile stress/strain curves for (**c**) GelMA/Bio-IL and (**d**) PEGDA/Bio-IL hydrogels at 80/20 polymer/Bio-IL ratio and various polymer concentrations. Error bars indicate standard error of the means, asterisks mark significance levels of p < 0.01 (**), and p < 0.001 (***)


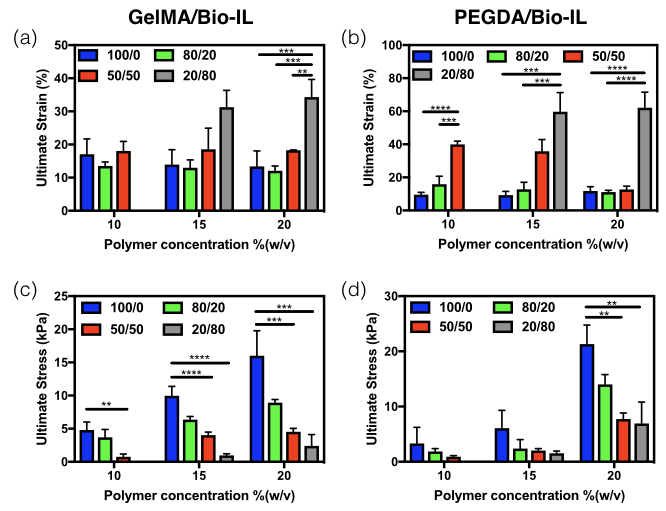


**Figure S4. Ultimate stress and ultimate strain of** **Bio-IL functionalized GelMA and PEGDA hydrogel crosslinked with visible light.** Ultimate strain values for (**a**) GelMA/Bio-IL and (**b**) PEGDA/Bio-IL hydrogels engineered by varying polymer concentration and polymer/Bio-IL ratios. Ultimate stress for (**c**) GelMA/Bio-IL and (**d**) PEGDA/Bio-IL hydrogels with varying polymer concentration and polymer/Bio-IL ratios (1% VC, 1.5% TEOA, 0.1 mM Eosin Y, and 120 s light exposure were used to form GelMA and PEGDA hydrogels). Error bars indicate standard error of the means, asterisks mark significance levels of p < 0.05 (*), p < 0.01 (**), and p < 0.001 (***).


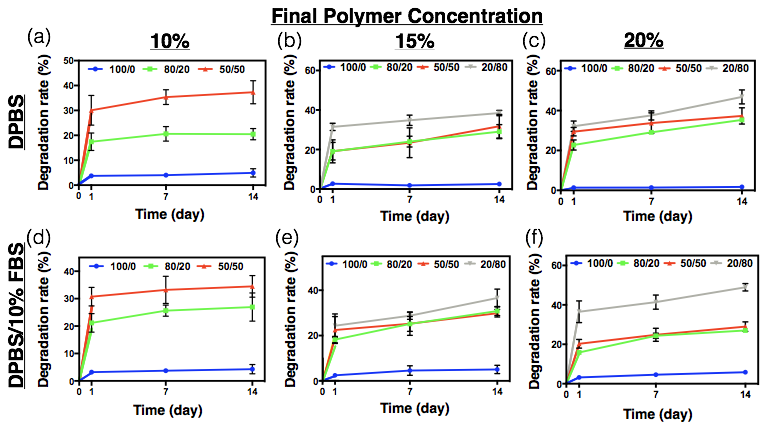


**Figure S5.** **Evaluation of the *in vitro* degradation of the GelMA/Bio-IL composite hydrogels.** The degradation rate of GelMA/Bio-IL hydrogels in DPBS using 10% (**a**), 15% (**b**), and 20% (**c**) final polymer concentration. Degradations rates were also determined for hydrogels in DPBS supplemented with 10% FBS using 10% (**d**), 15% (**e**), and 20% (**f**) final polymer concentration. In each of the concentrations tested, there were also four different ratios of GelMA/Bio-IL (1% VC, 1.5% TEOA, 0.1 mM Eosin Y, and 120 sec exposure to visible light were used to form the hydrogels).


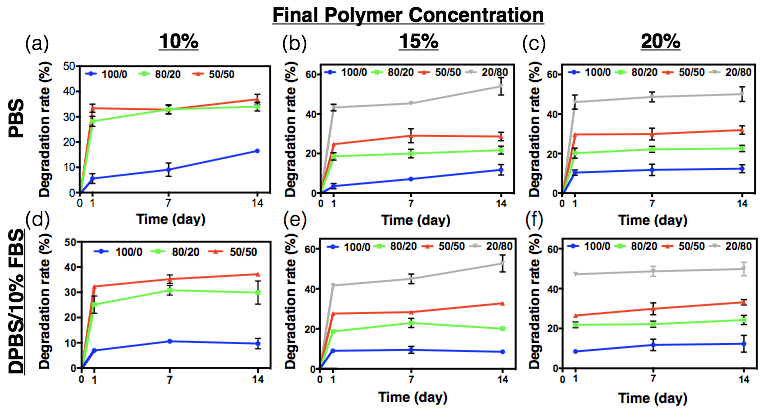


**Figure S6.** **Evaluation of the *in vitro* degradation of the PEGDA/Bio-IL composite hydrogels.** The degradation rate of PEGDA/Bio-IL hydrogels in DPBS using 10% (**a**), 15% (**b**), and 20% (**c**) final polymer concentration. Degradations rates were also determined for hydrogels in DPBS supplemented with 10% FBS using 10% (**d**), 15% (**e**), and 20% (**f**) final polymer concentration. In each of the concentrations tested, there were also four different ratios of PEGDA/Bio-IL (1% VC, 1.5% TEOA, 0.1 mM Eosin Y, and 120 sec exposure to visible light were used to form the hydrogels).


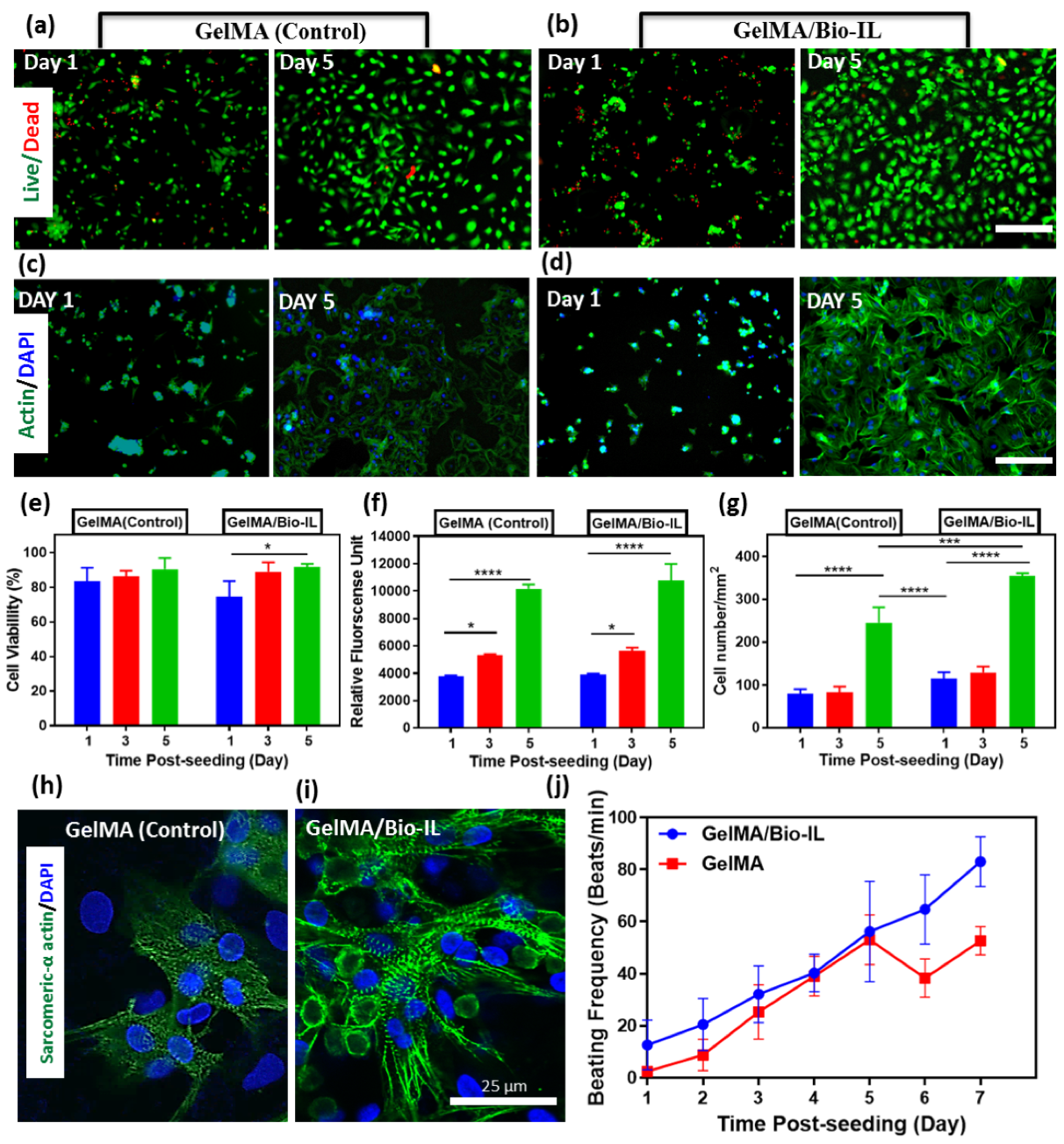


**Figure S7.** **2D *in vitro* studies using GelMA/Bio-IL hydrogels seeded with CMs.** Representative live/dead images from CMs cultured on (**a**) GelMA (control) and (**b**) GelMA/Bio-IL hydrogels on days 1 and 5 (scale bar = 200 μm). Representative F-Actin/DAPI stained images from CMs seeded on (**c**) GelMA and (**d**) GelMA/Bio-IL on days 1 and 5 (scale bar = 200 μm). (**e**) Cell viability quantification of CMs cultured on the hydrogels after 1, 3, and 5 days of culture. (**f**) Quantification of metabolic activity, relative fluorescence intensity (RFU) using PrestoBlue assay, 1, 3, and 5 days after seeding with CMs. **(g)** Quantification of cell number from DAPI-stained images of CMs seeded on the surface of GelMA and GelMA/Bio-IL hydrogels at days 1, 3, and 5 post-seeding. Immunofluorescent staining of sarcomeric α-actinin expressed by CMs seeded on GelMA **(h)** and GelMA/Bio-IL **(i)** hydrogels on day 7 (scale bar = 25 μm). (**j**) Characterization of synchronous contraction of CMs seeded on GelMA and GelMA/Bio-IL hydrogels over 7 days of culture. (*p < 0.05, **p < 0.01, ***p < 0.001 and ****p < 0.0001).
